# Transcriptome Profiles of Human Visceral Adipocytes in Obesity and Colorectal Cancer Unravel the Effects of Body Mass Index and Polyunsaturated Fatty Acids on Genes and Biological Processes Related to Tumorigenesis

**DOI:** 10.3389/fimmu.2019.00265

**Published:** 2019-02-19

**Authors:** Manuela Del Cornò, Antonella Baldassarre, Enrica Calura, Lucia Conti, Paolo Martini, Chiara Romualdi, Rosaria Varì, Beatrice Scazzocchio, Massimo D'Archivio, Andrea Masotti, Sandra Gessani

**Affiliations:** ^1^Center for Gender-Specific Medicine, Istituto Superiore di Sanità, Rome, Italy; ^2^Research Laboratories, Bambino Gesù Children's Hospital-IRCCS, Rome, Italy; ^3^Department of Biology, University of Padua, Padua, Italy

**Keywords:** obesity, body mass index, colorectal cancer, adipocyte, fatty acid, transcriptome, RNASeq

## Abstract

Obesity, a low-grade inflammatory condition, represents a major risk factor for the development of several pathologies including colorectal cancer (CRC). Although the adipose tissue inflammatory state is now recognized as a key player in obesity-associated morbidities, the underlying biological processes are complex and not yet precisely defined. To this end, we analyzed transcriptome profiles of human visceral adipocytes from lean and obese subjects affected or not by CRC by RNA sequencing (*n* = 6 subjects/category), and validated selected modulated genes by real-time qPCR. We report that obesity and CRC, conditions characterized by the common denominator of inflammation, promote changes in the transcriptional program of adipocytes mostly involving pathways and biological processes linked to extracellular matrix remodeling, and metabolism of pyruvate, lipids and glucose. Interestingly, although the transcriptome of adipocytes shows several alterations that are common to both disorders, some modifications are unique under obesity (e.g., pathways associated with inflammation) and CRC (e.g., TGFβ signaling and extracellular matrix remodeling) and are influenced by the body mass index (e.g., processes related to cell adhesion, angiogenesis, as well as metabolism). Indeed, cancer-induced transcriptional program is deeply affected by obesity, with adipocytes from obese individuals exhibiting a more complex response to the tumor. We also report that *in vitro* exposure of adipocytes to ω3 and ω6 polyunsaturated fatty acids (PUFA) endowed with either anti- or pro-inflammatory properties, respectively, modulates the expression of genes involved in processes potentially relevant to carcinogenesis, as assessed by real-time qPCR. All together our results suggest that genes involved in pyruvate, glucose and lipid metabolism, fibrosis and inflammation are central in the transcriptional reprogramming of adipocytes occurring in obese and CRC-affected individuals, as well as in their response to PUFA exposure. Moreover, our results indicate that the transcriptional program of adipocytes is strongly influenced by the BMI status in CRC subjects. The dysregulation of these interrelated processes relevant for adipocyte functions may contribute to create more favorable conditions to tumor establishment or favor tumor progression, thus linking obesity and colorectal cancer.

## Introduction

The increase of obesity afflicting nowadays adults and children worldwide has become a major global health challenge (1). Obesity, as defined by an excessive accumulation of white adipose tissue (AT), is characterized by a chronic low-grade inflammatory status triggered by AT expansion and hypoxia. AT is recognized as a key endocrine organ that regulates many metabolic and immune processes and plays a central role in obesity-associated morbidities including colorectal cancer (CRC) (2, 3). This tissue is endowed with functional pleiotropism relying on its capacity to synthesize and secrete a large array of mediators including cytokines/chemokines, extracellular matrix proteins, hormones, growth and vasoactive factors that influence, either locally or systemically, a variety of physiological and pathological processes (4). A central event in the etiology of obesity is the AT dysfunctionality characterized by hypertrophy, exacerbated inflammation, impaired extracellular matrix (ECM) remodeling and fibrosis, altered vascular function and structure that strongly contribute to the onset of obesity-associated comorbidities, including several types of cancer (5).

Obesity is the result of a complex interaction of factors of different nature such as genetic, metabolic, behavioral and environmental ones. Of note, specific dietary patterns and obesity degree have been associated with increased or decreased CRC risk and epidemiological studies highlighted the importance of nutrients in cancer risk or prevention (6, 7). Food components able to modulate inflammation are of great interest in the prevention of obesity-related morbidities, including cancer, as the condition of chronic inflammation represents a main risk factor for tumors. Interestingly, dietary components as well as weight loss have been associated with changes in gene expression of AT (8). In this regard, the fatty acid (FA) profile of AT, in particular of polyunsaturated fatty acids (PUFA), closely reflects their dietary intake and may impact metabolic, inflammatory and immune processes (9). PUFA consist of two families (ω3 and ω6) able to modulate the inflammatory response (10). While ω3 PUFA have been described to attenuate inflammation, ω6 PUFA, with a few exceptions, are endowed with pro-inflammatory activity (11, 12).

The mechanisms underlying the detrimental link between obesity and cancer are still a matter of debate. In this respect, it has been postulated that this association may be due to the large spectrum of mediators that are produced by AT showing pro-inflammatory and cancer prone features. The altered expression of these molecules or adipocyte functional dysregulation promoted by obesity, play a critical role in the impairment of body homeostasis. Obesity-related metabolic alterations involved in carcinogenesis (i.e., the triggering of insulin resistance and of immune function alterations, cell proliferation, migration, angiogenesis and oxidative stress) may contribute to the initiation and progression of CRC (13). Several attempts have been made to investigate the molecular mechanisms involved in obesity that may create a more favorable environment to cancer establishment. Gene expression profiling has been instrumental to characterize biological pathways affected by either weight gain or loss. A number of transcriptomic studies based on microarray technology have been carried out on whole AT, mainly the subcutaneous AT (SAT), highlighting pathways modulated by obesity, specific nutrient-enriched diet or by weight loss (14–19). Only recently, the RNA sequencing (RNASeq) technology has been used to assess obesity-related modification of SAT transcriptome (20–22) but not to study that of purified adipocytes from visceral AT (VAT). Interestingly, accumulation of visceral fat correlates with metabolic syndrome (23) and represents a main risk factor for CRC (24).

In this study, we analyzed RNASeq expression profiles of human adipocytes purified from VAT of lean and obese subjects affected or not by CRC. This approach highlighted changes in adipocyte transcriptional program that are specifically associated with obesity or CRC, or shared by both conditions. We found that, the most relevant changes involved pathways and biological processes linked to ECM remodeling, pyruvate, lipid and glucose metabolism. Furthermore, we provided evidence that body mass index (BMI) has an impact on adipocyte cancer-specific transcriptional profile as well as on adipocyte response to exogenous PUFA exposure. Overall, these results add further evidence for a central role of AT dysfunction in obesity-associated morbidities such as cancer, suggesting that the deregulation of biological processes relevant for adipocyte functions may contribute to create a more favorable environment to tumor establishment.

## Methods

### Patient and Sample Collection

Human visceral adipose tissue was collected from age-matched lean and obese subjects undergoing abdominal surgery or laparoscopy for benign (i.e., gallbladder disease without icterus, umbilical hernia, and uterine fibromatosis) or CRC conditions (histologically proved primary colon adenocarcinoma, stage TNM 0–III). The exclusion's criteria were: clinical evidence of active infection, recent (within 14 days) use of antibiotics/anti-inflammatory drugs, pregnancy, hormonal therapies, severe mental illness, autoimmune diseases, family history of cancer, other neoplastic diseases. In the normal weight group, the BMI range was 20–25 Kg/m^2^. In the obese group the BMI was ≥ 30 Kg/m^2^, and waist circumference > 95 cm for men and > 80 cm for women. The number of subjects for RNASeq analysis was six/category (five for the ObCRC group), or ranged from five to nine samples for real-time quantitative PCR (qPCR) analysis, as biological replicates, in addition to technical ones, were analyzed for some categories. There were no significant differences in the age between obese and lean control subjects (participants were on average 39.7 years old). However, the age was significantly higher for the CRC patients (participants were on average 65.3 years old) included in both the RNASeq and real-time qPCR analyses. The anthropometric characteristics of subjects enrolled in the study are reported in Table 1.

**Table 1:** Anthropometric characteristics of subjects included in the study.

	**Lean/Nw**		**Obese/Ob**		**P obesity**		**P cancer**	
	**Non-CRC**	**CRC**	**Non-CRC**	**CRC**	**Nw vs. Ob**	**NwCRC vs. ObCRC**	**Nw vs. NwCRC**	**ObCRC vs. Ob**
N (Male/Female)	6 (0M, 6F)	9 (4M, 5F)	8 (3M, 5F)	9 (7M, 2F)	–	–	–	–
Age	41.6 ± 3.4	62.9 ± 5.4	37.8 ± 6	67.7 ± 4.5	0.3603	0.4147	0.0027	< 0.0001
Body weight (Kg)	57.3 ± 2.9	65.9 ± 5.4	110.7 ± 12.6	96.3 ± 4.5	< 0.0001	0.0025	0.3887	0.1515
BMI (Kg/m^2^)	22 ± 1	23.5 ± 1	37.8 ± 2.1	33.8 ± 3.2	< 0.0001	< 0.0001	0.4719	0.1190

*Data are mean ± SEM. BMI, body mass index. Statistical differences were analyzed by one-way ANOVA followed by Bonferroni post-hoc test*.

### Adipocyte Isolation and Culture

AT sampling was performed as previously described (25). Fifteen to forty grams of AT biopsies were microdissected, rinsed several times in 0.9% NaCl, and digested with 5 ml of Krebs-Ringer solution (0.12 M NaCl, 4.7 M KCl, 2.5 mM CaCl_2_, 1.2 mM MgSO_4_, 1.2 mM KH_2_PO_4_) containing 20 mM HEPES pH 7.4, 3.5% BSA fatty acid-free, 200 nM adenosine, 2 mM glucose and collagenase (type 1) for 1 h (1 mg/g adipose tissue) at 37°C in shaking water bath. After collagenase digestion the adipocytes were isolated and cultured in low-glucose Dulbecco's modified Eagle's medium [1,000 mg/L D-(+)-glucose]. In some experiments (3 subjects/category), adipocytes were stimulated for 18 h with docosahexaenoic acid (DHA) (Sigma Aldrich) or arachidonic acid (AA) (Cayman Chemical Company), as we previously described (26). DHA and AA were dissolved under a nitrogen atmosphere in 100% ethanol to make 10 mM stock solutions, which were stored at −20°C. Stock solutions were diluted in culture media prior to the cell treatment. Final concentration of ethanol in treated cells was <0.1%. To define the lowest effective concentration of DHA and AA able to modulate adipocyte activities, we carried out preliminary experiments, incubating the isolated adipocytes with different concentration of DHA (5–50 μM) and AA (1–25 μM) for different periods of time (6–24 h). On the basis of the data obtained (not shown), the experiments were carried out incubating the adipocytes with 10 μM DHA and 5 μM AA for 18 h, respectively.

### mRNA Preparation and Sequencing

Total RNA was isolated with Total RNA Purification Plus Kit (Norgen Biotek, Canada). RNA quality and quantity was assessed by Agilent 2,100 Bioanalyzer and samples stored at −80°C until use. Total RNA (2 μg) was used to prepare the library for Illumina sequencing. Single-end reads (60 M per sample) were produced by Illumina HiSeq 2000.

### RNASeq Data Preprocessing and Differential Expression Analysis

The transcriptome reconstruction was performed by the Tuxedo protocol. Reads were mapped to the human genome (hg38.p2 version) using HISAT2 (27). Human genome and annotations of reference genes and transcripts (Ensembl 79) were provided as input data. Alignments were then elaborated by StringTie (28), which assembled and quantified the transcripts in each sample. Subsequently, the sample-specific assembled transcriptomes were merged together by a dedicated StringTie module, which created a uniform set of transcripts for all samples. The Gffcompare program was used to compare the genes and transcripts to the existing annotations (28). We used RSEM to estimate transcript abundances. Transcripts with <10 counts in at least 60% of samples per group of subjects were filtered out. Filtered raw counts matrix can be found in Supplemental Data 1. Differential expression (pairwise comparisons) was computed by edgeR (version 3.18.1) from raw counts following authors' instructions and the batch effect correction was applied by RuvSeq (29), using standard procedure with factors of unwanted variation optimized to *k* = 7. Multiple testing controlling procedure was applied following Benjamini & Hochberg method hereafter referred as False Discovery Rate (FDR). Transcripts with a corrected *p*-value (FDR) ≤ 0.05 and log2(Fold Change) ≥ |2| were considered differentially expressed.

The datasets generated for this study can be found in the SRA repository (SRA accession number: PRJNA508473).

### Real-Time qPCR Validation of Differentially Expressed Transcripts

Gene expression profiling was performed using a custom-made Taqman® low-density array (TLDA) to analyze 96 selected candidate genes (including four housekeeping genes) that were found in obese and CRC subjects by RNASeq analysis. Candidate genes were selected among those belonging to the enriched GO categories and pathways as well as on the basis of their relevance to obesity- and CRC-linked processes. The synthesis of cDNA from 1 μg of total RNA was carried out in 20 μL reaction volume using the High Capacity cDNA kit (Applied Biosystems) following the manufacturer's instructions. The reverse transcription conditions were as follows: 10 min at 25°C, 120 min at 37°C, and 5 sec at 85°C. cDNA was mixed with 2 × TaqMan Universal PCR Master Mix (Applied Biosystems), loaded on the TLDA card, and run on an QuantStudio 12 K Flex Real-Time PCR System (Applied Biosystems) following the manufacturer's instructions. Gene expression values were normalized to the expression of GUSB (selected house-keeping gene). GUSB has been selected as endogenous control to normalize data owing to its lowest standard deviation (0.97) compared to ACTB (1.83), GAPDH (1.82) and 18S (1.98), in control lean subjects. For each sample, the relative gene expression level was determined according to the 2 ^−ΔΔ*CT*^ method.

### Functional Analyses

To assess the function of differentially expressed transcripts (DET), DAVID (Database for Annotation, Visualization and Integrated Discovery) bioinformatic resource (30) was employed to classify them into cellular component, biological process and molecular function Gene Ontology (GO) categories. Significantly enriched pathways of these DET were then determined by KEGG (Kyoto Encyclopedia of Genes and Genomes) database. Functional interactions between genes were predicted by the GeneMANIA webserver (http://www.genemania.org) (31). Given a query gene list, functionally similar genes were found using a wealth of genomics and proteomics data by weighting each functional genomic dataset according to its predictive value for the query.

### Statistical Analysis

Statistical comparisons of means from several experiments was performed between the various categories of subjects by one-way analysis of variance (ANOVA) with either Bonferroni *post-hoc* tests by using GraphPad Prism 5 software for the analysis of real-time qPCR data or the function p.adjust (package stats) in R Bioconductor, that was employed to obtain corrected FDR values for functional analysis of data. Differences were considered statistically significant when *p*-values were ≤ 0.05. Statistical significance is indicated with ^*^p ≤ 0.05, ^**^p ≤ 0.005 and ^***^p ≤ 0.0005.

## Results

### Differential Expression of Adipocyte Transcripts in Obesity and CRC

**Figure 1:**
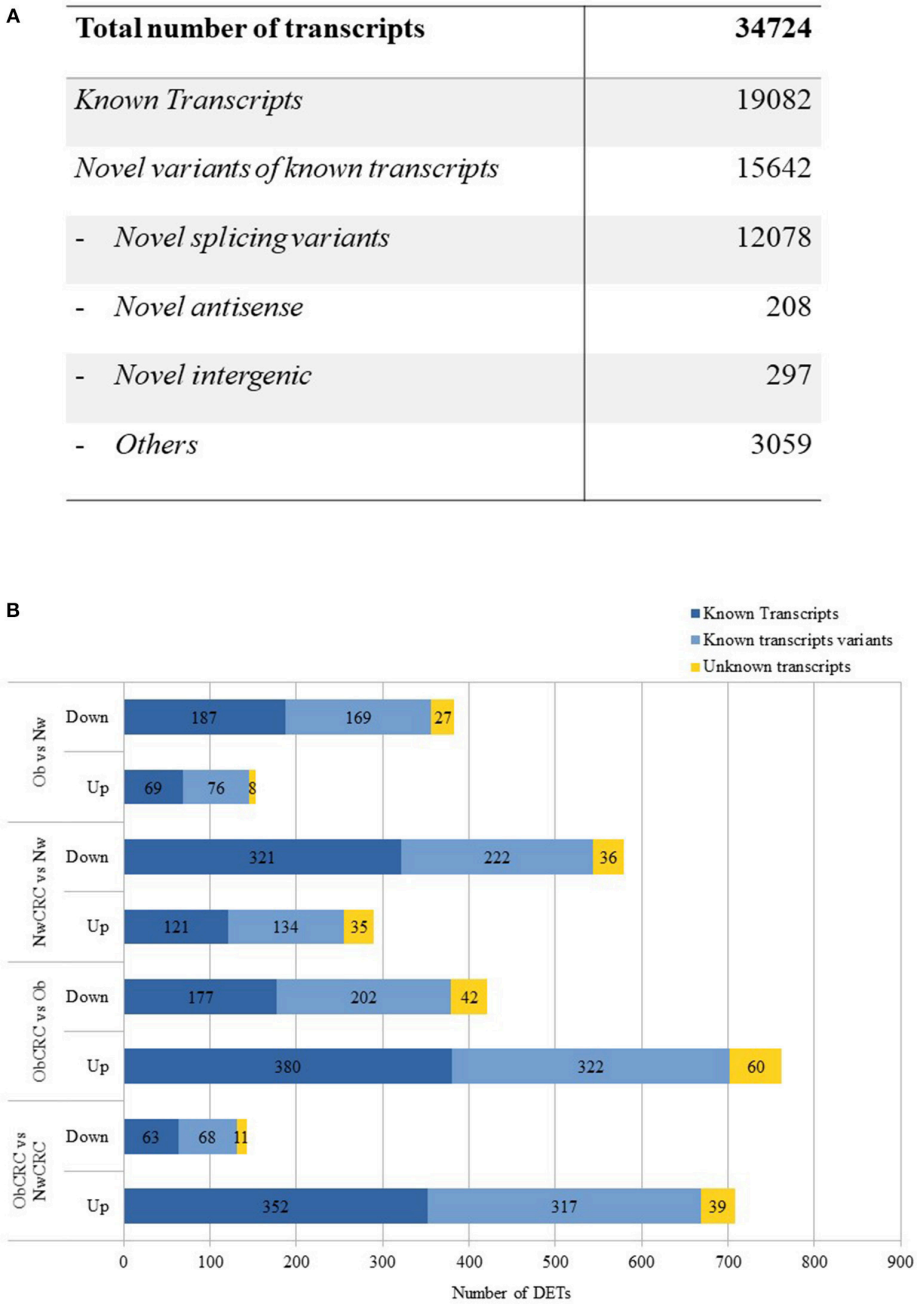
RNASeq analysis. **(A)** numbers of different types of transcripts in the reconstructed transcriptome. **(B)** up- and down-regulated differentially expressed transcripts obtained by the comparison of healthy lean (Nw), obese (Ob) and CRC-affected groups [*n* = 6 subjects/category with the exception of obese affected by CRC (ObCRC) group in which *n* = 5]. Differentially expressed transcripts are divided in: known transcripts, variants of known transcripts and unknown transcripts.

The transcriptome of human adipocytes isolated from visceral AT biopsies from control lean (normal weight, Nw) and obese (Ob) subjects, affected or not by CRC (NwCRC and ObCRC, respectively), was obtained using RNASeq analysis. Along with the known transcripts, the analysis detected also many variants of known transcripts, including novel splicing variants of know transcripts and a small number of totally new elements (unknown intergenic and novel antisense). After filtering weakly expressed transcripts, 34,724 transcripts were reconstructed of which 19,082 are known and 15,642 are variants of known transcripts, i.e., differing from the corresponding reference transcripts for at least one base in at least one splice junction (Figure 1A). This proportion is largely expected from a total RNA sequencing in which in addition to the mature transcripts, unspliced or partially unspliced transcripts are also measured.

DET across the four categories of subjects may provide clues on the mechanisms by which obesity can favor CRC development as well as on how obesity-associated carcinogenesis differs from that of lean individuals. To this aim, we compared the gene expression profiles in the four categories of subjects, focusing on the known transcripts (Figure 1B). Variants of known as well as totally unknown transcripts were not considered for the analyses hereafter. RNASeq analysis revealed 536 DET when comparing adipocyte profiles from Ob and Nw subjects. Among these genes, 153 were up-regulated (69 known transcripts) while 383 were down-regulated (187 known transcripts), highlighting a different transcriptional behavior of adipocytes under these two conditions. 869 DET were found in the adipocytes of NwCRC compared to Nw subjects, of which 290 were up-regulated (121 known transcripts), while 579 (321 known transcripts) were down-regulated. Conversely, a similar comparison of ObCRC with Ob category of subjects revealed a higher number (1183, 557 known transcripts) of genes deregulated in the adipocytes of ObCRC with respect to Ob subject counterpart suggesting an interplay between obesity- and CRC-induced modulations of adipocyte transcriptional program. Furthermore, in contrast to what observed for NwCRC subjects, the majority of transcripts (762 vs. 290, of which 380 were known transcripts) were up-regulated while a smaller number was down-regulated (421 vs. 579, of which 177 were known transcripts) in ObCRC patients.

Finally, to the end of further defining the influence of BMI on the changes of adipocyte transcriptional program in cancer, we directly compared the transcriptome of ObCRC with that of NwCRC patients. We found 850 DET of which 708 were up-regulated (352 known transcripts), while only 142 were down-regulated (63 known transcripts), confirming that CRC status is associated with a different adipocyte transcriptional profile in lean and obese subjects. The complete list of DET in the different comparisons, along with additional information such as genomic position, strand and transcript type when available, are shown in Supplemental Data 2. Volcano plots showing the magnitude and the significance of the expression changes are also provided in Supplemental Data 3.

### Gene Ontology and KEGG Pathway Enrichment Analysis of DET

GO term enrichment and KEGG Pathway analysis by DAVID bioinformatics tool was used to explore biological processes, molecular functions, cellular components and pathways significantly enriched (FDR ≤ 0.05) among the DET, focusing on those most relevant to obesity and cancer. The complete list of GO categories and KEGG pathways enriched in the different comparisons is shown in Supplemental Data 4.

**Figure 2:**
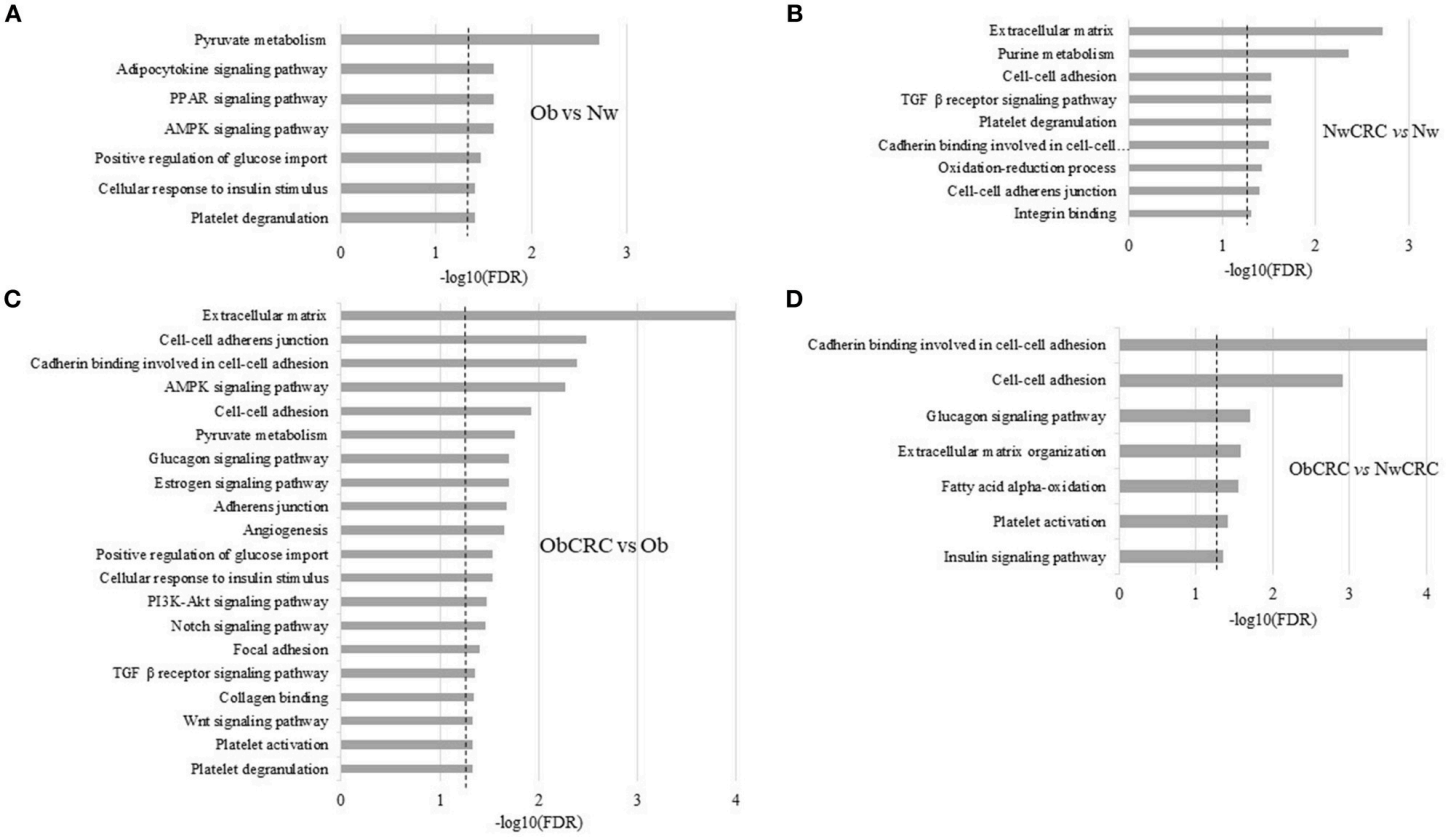
Functional analysis of differentially expressed transcripts. Histograms represent significant (FDR ≤ 0.05) GO terms and KEGG pathways of genes modulated by obesity (Ob) vs. normal weight (Nw) **(A)**, normal weight affected by CRC (NwCRC) vs. normal weight (Nw) **(B)**, obese affected by CRC (ObCRC) vs. obese (Ob) **(C)**, and in obese affected by CRC (ObCRC) vs. normal weight affected by CRC (NwCRC) individuals **(D)**. FDR have been reported on X axis and dotted line represent the FDR cut-off (≤ 0.05).

As shown in Figure 2A, pathways associated with cellular energy metabolism (e.g., pyruvate, glucose and FA metabolism) as well as with inflammation were deregulated in obese with respect to lean control subjects. The most enriched category “*Pyruvate metabolism”* reflected the modulation of genes encoding for enzymes, mostly dehydrogenases, involved in the interconversion of pyruvate and lactate (LDHB, LDHD), detoxification of aldehydes generated by alcohol and lipid peroxidation (ALDH7A1, ALDH3A2), and FA oxidation (ACACB), while in the category “*PPAR signaling pathway”* were genes involved in FA and lipid transport (SLC27A6, CD36 and SCP2) and peroxisomal lipid metabolism (ACOX3). Among genes in the category “*AMPK signaling pathway”* were LEP, ADIPOR2, CD36, and IGF1, also recapitulated in the category “*Adipocytokine signaling pathway,”* while the categories “*Positive regulation of glucose import”* and “*Cellular response to insulin stimulus”* shared the genes CAPN10 and RHOQ. Finally, the “*Platelet degranulation”* category included among others CD9, CALM3 and ECM1.

In NwCRC group (Figure 2B) the three most enriched categories with respect to the lean counterpart were “*Extracellular matrix,” “Purine metabolism”* and “*TGF*β *receptor signaling pathway”* reflecting the modulation of genes involved in ECM remodeling, such as matrix metalloproteinases or structural components (ADAMTSL4, ECM1, LAMB2 and LMNA), cyclic nucleotides, ATP metabolism (PDE1A and 7A, AK2 and 3, AMPD2, ATPR, ATP5A1), and several members of TGFβ receptor signaling pathway (TGFB1 and 3, TGFBR2, LTBP2, SMAD6). Genes involved in metabolic processes (LDHD, ALDH1A2, ALDH7A1, ALDH3A2) drove the enriched category “*Oxidation-reduction process*.” Categories of cellular components, biological processes and molecular functions related to cell-cell interaction and communication *(“Cell-cell adhesion,” “Cadherin binding involved in cell-cell adhesion,” “Cell-cell adherens junction,” “Integrin binding”)* were also found enriched, driven by genes contributing to the obesity-associated remodeling of AT (19) such as ECM2, encoding for an ECM protein predominantly expressed in AT, and ADAM15, encoding for a transmembrane glycoprotein involved in cell adhesion and cytokine/adhesion molecule processing were also among genes enriching this category. “*Platelet degranulation”* was further enriched and included common (CD9, ECM1 and ACTN1) and unique (ISLR, PSAP, TGFB3, SERPINA3, TTN) genes with respect to Ob group.

In keeping with the observation that patient BMI status may affect adipocyte transcriptional program in CRC condition, DAVID analysis of those DET arising from the comparison of ObCRC with the Ob group (Figure 2C) revealed that a higher number of biological processes are modulated when CRC develops in Ob individuals. In particular, shared processes related to cell adhesion were further enriched in ObCRC with respect to NwCRC patients, involving either common or unique genes, such as collagens (COL4A2, COL12A1 and COL18A1) or integrins (ITGAV, ITGA8), as well as additional related categories of processes like “*Collagen binding”* and “*Focal adhesion”* that includes genes involved in tissue remodeling and fibrosis. Among others were DDR1, PCOLCE2 and SPARC genes. Other categories, although remaining similarly represented (e.g., “*TGF*β *receptor signaling pathway”* and “*AMPK signaling pathway*”), differed for the modulation of SREBF1, encoding for a transcription factor that regulates the expression of critical elements of glucose uptake and lipogenic pathways in AT. Furthermore, a number of categories not found to be enriched in NwCRC with respect to Nw individuals were instead modulated in ObCRC with respect to Ob subjects including “*Glucagon signaling pathway,” “Estrogen signaling pathway,” “PI3K-Akt signaling pathway,” “Notch signaling pathway,” “Platelet activation,”* “*Angiogenesis*,” and “*Wnt signaling pathway.”* In these enriched categories genes involved in FA synthesis (ACACA), gluconeogenesis (PCK2) and insulin signaling (AKT2) were included. Other transcripts deregulated in ObCRC group compared to Ob subjects are COL4A2, ANXA2 and ACVRL1 involved in angiogenesis, GNAS and FYN associated with tumorigenesis. The biological processes “*Platelet degranulation,” “Positive regulation of glucose import”* and “*Cellular response to insulin stimulus,”* already found modulated in obesity (PDGFB, SERPING1 ADIPOQ, PIK3R1, IGF1) were further enriched in ObCRC with respect to Ob individuals.

Finally, to the end of further defining the influence of BMI on adipocyte transcriptional program of CRC patients, ObCRC and NwCRC transcriptomes were directly compared (Figure 2D). The results of this analysis revealed that a number of pathways already enriched in the comparison of CRC patients with their non-CRC counterparts (Figures 2B,C) (“*Cell-to-cell adhesion,” “Cadherin binding involved in cell-cell adhesion,” “Extracellular matrix organization,” “Glucagon signaling pathway,” “Platelet activation”*) were found further enriched when directly comparing ObCRC with NwCRC patients, highlighting a stronger dysregulation of these processes in the obese background. Furthermore, some categories of genes associated to processes linked to adipocyte metabolism *(“Fatty acid alpha-oxidation,”* including the gene SLC27A2 and ALDH3A2; “*Insulin signaling pathway,”* including the gene SREBF1 and AKT2) were selectively modulated in ObCRC with respect to NwCRC patients.

### Common or Unique Genes and Biological Processes Are Modulated in Obesity and CRC

**Figure 3:**
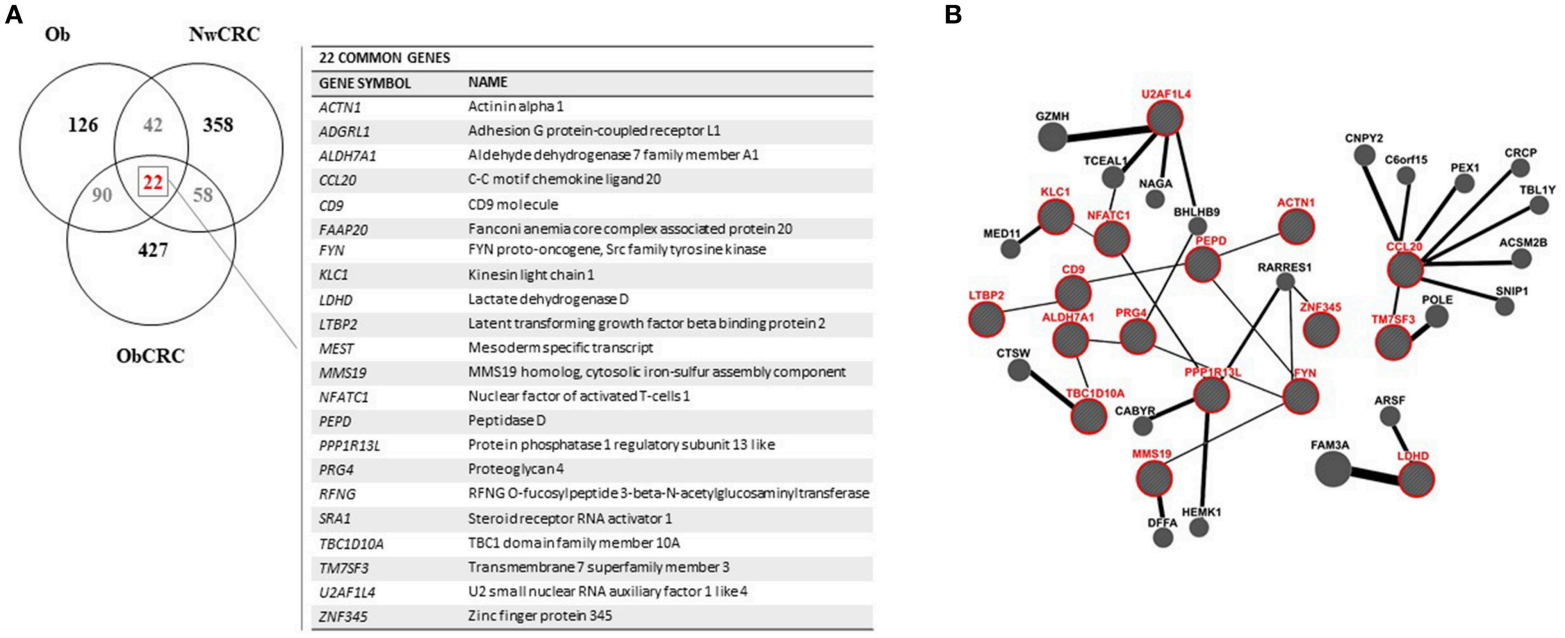
Analysis of genes shared by obese and CRC-affected individuals. **(A)** venn diagram showing unique or shared genes resulting by the comparison of DET from all pathological conditions vs. healthy lean subjects. Each comparison is represented by a circle. The numbers in the region of the overlapping circles indicate the genes that are expressed in two or more conditions. The complete list of the 22 genes shared by obesity and CRC, regardless of BMI, is shown on the right. **(B)** gene network functional interactions obtained by GeneMANIA. The network was constructed starting from the 22 genes common to all pathological conditions (in red) that are functionally connected with the main interactors.

Obesity and CRC are multifactorial disorders/diseases characterized by an inflammatory status. In obesity, visceral AT-associated inflammation is considered an important determinant in the development of obesity-related morbidities including CRC (32). On the other end, inflammation is a hallmark of cancer representing a condition that can favor carcinogenesis or be a consequence of the tumor onset/treatment (33). To identify genes/processes modulated in obesity- and/or cancer-related inflammation, Venn diagrams were used to assess the number of DET and their overlap/uniqueness among the experimental groups of Ob, NwCRC and ObCRC subjects. As shown in Figure 3A, the comparison of the transcriptomes of Ob and CRC groups with that of non-CRC controls revealed that the majority of DET were uniquely modulated in the different categories (126 in obesity, 358 and 427 in CRC affected lean and obese patients, respectively), 90 were obesity-specific since shared by Ob and ObCRC groups, while 58 were cancer-specific as common to CRC patients regardless the BMI. Lastly, 42 genes were common to Ob and NwCRC subjects suggesting that they might be related to the inflammatory condition shared by these groups of subjects. Interestingly, only 22 genes, listed in Figure 3A, were common to all groups. Among them are genes involved in cell adhesion/migration processes (PEPD), migratory potential of cancer cells (ACTN1), ECM (LTBP2, PRG4, ADGRL1, CD9), or in metabolic processes (LDHD, ALDH7A1), as well as proto-oncogenes or their ligands/inhibitors (FYN, PPP1R13L).

To determine the interrelationship among this core set of genes, we applied GeneMANIA network analysis, the results of which are displayed in Figure 3B. This analysis emphasized the presence of many sub-networks. In particular, we found two sub-networks made by 5 interacting genes (i.e., LTBP2-CD9-PEPD-FYN-MMS19 and TBC1D10A-ALDH7A1-PRG4-FYN-MMS19), another one with 4 genes (i.e., LTBP2-CD9-PEPD-ACTN1) and the smallest with 3 interacting genes (i.e., PPP1R13L-NFATC1-KLC1). Furthermore, additional interconnected genes were predicted by GeneMania (BioGRID data) to be functionally related to the identified sub-networks. The identification of a network of functional interactions among the 22 genes shared by all pathological conditions, suggested that these genes (and their interactors) could be involved in pathogenic processes relevant for obesity and CRC.

**Figure 4:**
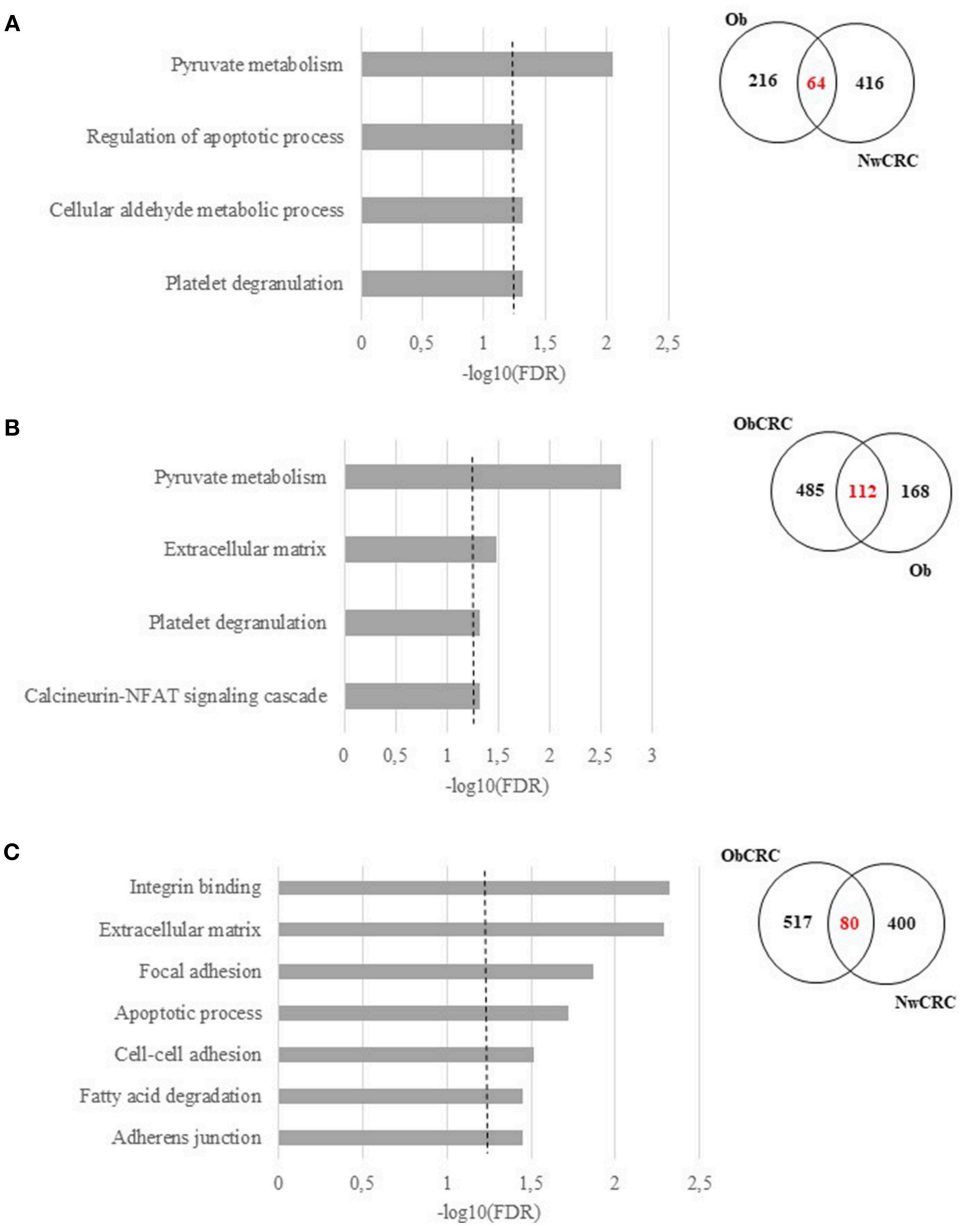
Functional analysis of differentially expressed transcripts shared among obesity and/or CRC. Significant GO terms and KEGG Pathway enrichment analysis (FDR ≤ 0.05) of shared genes and Venn diagrams resulting by the overlap between, **(A)** obese (Ob) and normal weight affected by CRC (NwCRC) groups compared to normal weight (Nw) group. **(B)** obese (Ob) compared to normal weight (Nw) or obese affected by CRC (ObCRC) groups. **(C)** CRC groups (NwCRC or ObCRC) compared to their non-CRC counterparts (Nw or Ob). FDR have been reported on X axis and dotted line represent the FDR cut-off (≤ 0.05).

To further highlight similarities or differences between obesity and CRC conditions, we performed GO and KEGG Pathway enrichment analyses on the genes obtained from the comparisons (i.e. Ob/ Nw vs. NwCRC/Nw; Ob/Nw vs. ObCRC/Ob; ObCRC/Ob vs. NwCRC/Nw) shown in Figure 3A, focusing on the overlapping regions. Results are listed in Supplemental Data 5 and summarized in Figure 4. Based on the hypothesis that dysfunction of AT in obesity may be a condition favoring CRC, we searched for candidate genes and biological processes that may contribute to the obesity-CRC link. To this aim GO and KEGG pathway analysis of the 64 genes shared by Ob and NwCRC condition with respect to the healthy lean group (Figure 4A), revealed a number of processes modulated in both obesity and cancer condition, including: “*Pyruvate metabolism*,” “*Cellular aldehyde metabolic process*,” “*Platelet degranulation*,” and “*Regulation of apoptotic process*.” These processes likely include obesity-associated genes that may favor cancer establishment. Similarly, the genes shared by Ob and ObCRC groups (112 DET) with respect to their lean or obese controls (Figure 4B) belong to “*Pyruvate metabolism*” and “*Platelet degranulation*” categories, and to additional biological processes, including: “*Extracellular matrix*,” that could contribute to the AT fibrotic process observed in obesity, and “*Calcineurin-NFAT signaling cascade*,” an important regulator of immune system and inflammatory responses.

Finally, to go deeper in understanding the impact of BMI on the transcriptional profile of adipocytes under CRC condition, the same analysis was performed on DET [80] shared by ObCRC and NwCRC patients with respect to their non CRC counterparts (Figure 4C). The majority of them encoded proteins mainly belonging to ECM and cell adhesion categories *(“Extracellular matrix,” “Adherens junction,” “Cell-cell adhesion,” “Focal adhesion,” “Integrin binding,” “Cadherin binding involved in cell-cell adhesion”)*. Moreover, categories related to lipid metabolism *(“Fatty acid degradation”)* and apoptosis *(“Apoptotic process”)* were also found to be enriched. The genes associated to the selected GO categories and pathways shown in Figure 4, and potentially involved in the complex relationship between adiposity and CRC, are listed in Supplemental Data 6.

### Gene Expression Validation by Using Real-Time Quantitative PCR

**Figure 5:**
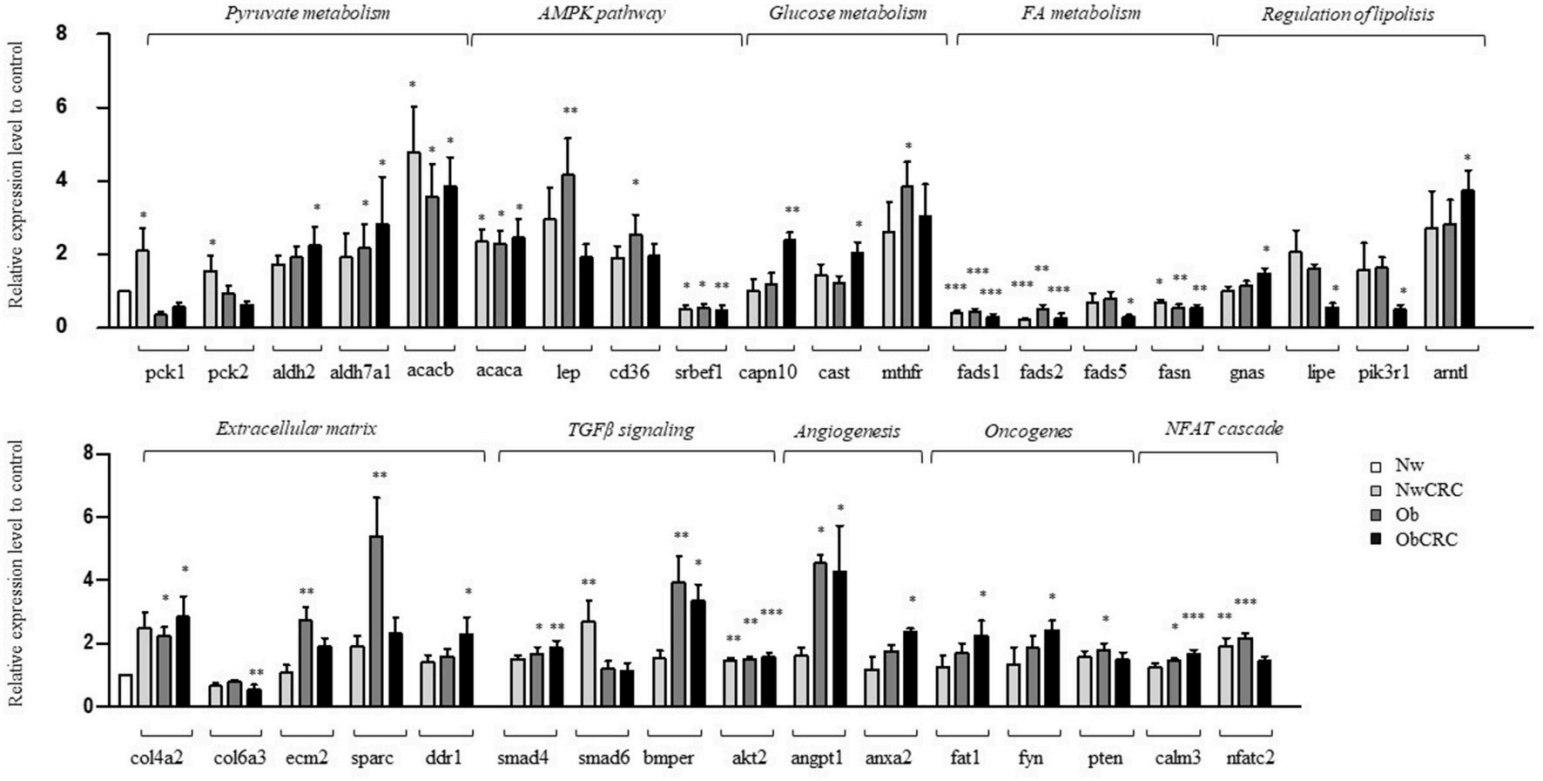
Validation by real-time qPCR of selected genes. Real-time qPCR analysis on adipocytes from normal weight (Nw), obese (Ob), normal weight and obese individuals affected by CRC (NwCRC and ObCRC, respectively). Selected genes involved in adipocyte metabolism or linked to tumorigenesis, were quantified and normalized to Normal weight control. ^*^p ≤ 0.05, ^**^p ≤ 0.005, and ^***^p ≤ 0.0005.

In this study, we used RNASeq to define the general landscape of processes occurring in the different categories of subjects. As a further step to validate the observed transcript modulations, we performed real-time qPCR by selecting 92 genes among the identified DET. Candidate genes were selected among those belonging to the enriched GO categories and pathways, shared or unique, in obese and/or CRC conditions, as well as on the basis of their relevance in metabolism, lipogenesis, fibrosis and AT microenvironment. The complete list of genes and probes for real-time qPCR analysis is shown in Supplemental Data 7. Out of 92 analyzed genes, 36 were found to be significantly modulated by real-time qPCR in adipocytes from the different categories of subjects (obese and CRC affected) with respect to healthy lean subjects (Figure 5). Although the correspondence between gene expression data achieved by the two different methods was not complete, in most cases data obtained by real-time qPCR had a similar directional pattern. Within this set of genes, the modulations observed in RNASeq analysis that were not confirmed by real-time qPCR ranged from 0 to 11% depending on the comparison, while an opposite modulation was observed in 5,5 to 16 % of genes, depending on the comparison. As shown in Figure 5, the modulated genes were found to be either specific for obesity or CRC or shared by both conditions, similarly to what observed for the biological processes. Among the latter (Figure 5, upper panel) we found genes involved in lipid metabolism (i.e., FA metabolism and AMPK pathway). Interestingly, *de novo* synthesis of FA is a major event in the metabolic transformation that leads to cancer (34). The expression of genes of the FA desaturase (FADS1 and FADS2) and the FA synthase (FASN) families was down-regulated in both obese and CRC affected subjects regardless of BMI, with the exception of FADS5 expression that is reduced in the ObCRC group only. In addition, ACACB gene encoding for acetyl-CoA carboxylase 2, which allows the acetyl-CoA/malonyl-CoA conversion, rate-limiting step in FA synthesis, was over-expressed in all groups. Within the AMPK signaling pathway, SREBF1, coding for sterol regulatory element binding protein-1, the master regulator of FA and triacylglycerol synthesis, as well as ACACA, another key lipogenic enzyme, were also found to be up- and down-regulated, respectively, in all groups. The AMPK pathway also includes CD36, an important regulator of cell adhesion and FA transport, and LEP gene, a major regulator of energy homeostasis, whose transcription, as expected, was up-regulated in obesity. Similarly, genes involved in processes related to ECM remodeling, angiogenesis, pyruvate metabolism and TGFβ signaling, potentially linked to tumorigenesis, were also predominantly regulated under obesity. As shown in Figure 5 (lower panel), the expression of the ECM components ECM2, encoding for an ECM protein predominantly expressed in AT, and SPARC, a key regulator of collagen-mediated signaling, were up-regulated in Ob subjects, COL4A2 in both Ob and ObCRC conditions, while another collagen family member, COL6A3, and the collagen-dependent receptor tyrosine kinase DDR1, mediating cell adhesion to collagen, were specifically down- and up-modulated, respectively, in CRC-affected obese subjects. Moreover, deregulation of the TGFβ signaling pathway was confirmed by the up-regulation of SMAD4 and BMPER in obese groups, and AKT2 in all pathological conditions. Finally, genes implicated in the regulation of angiogenesis (ANGPT1, ANXA2), pyruvate and glucose metabolism (aldehyde dehydrogenase genes ALDH2 and ALDH7A1, and MTHFR) and calcineurin-NFAT signaling cascade (CALM3), as well as the tumor suppressor PTEN, were also found up-regulated in obese condition (Figure 5, upper and lower panels). In keeping with the results of GO analysis, some of the validated genes were found selectively regulated in obese subjects affected by CRC. Among them, genes of the glucose metabolism, such as CAPN10, encoding for calpain, a calcium-sensitive cysteine protease associated with type 2 diabetes, and its inhibitor CAST, involved in obesity-induced AT inflammation, were up-regulated in this subject group. The ObCRC group is also characterized by an altered expression of a number of genes involved in lipid metabolism (GNAS, LIPE, PIK3R1, ARNTL) as well as of genes which have a documented role in carcinogenesis, like the atypical cadherin FAT1 and the proto-oncogene FYN (Figure 5, lower panel). On the other hand, genes relevant for pyruvate metabolism, such as the phosphoenolpyruvate carboxykinase genes PCK1 and 2, main control points for the regulation of gluconeogenesis, as well as SMAD6, involved in TGFβ signaling, were specifically up-regulated in adipocytes from lean CRC patients. In addition to the above described gene expression alterations common to all the pathological conditions, lean CRC patients were also found to share with the Ob group the up-regulation of NFATC2 involved in calcineurin-NFAT signaling cascade.

### ω3 and ω6 PUFA Differentially Affect Gene Expression in Adipocytes From Lean and Obese Subjects

Several dietary factors, in particular FA have been previously associated with changes in gene expression in AT (35) and dietary patterns/diet components have been linked with a higher or lower cancer risk (36, 37). Based on the observation that obesity deregulates genes involved in FA metabolisms and on our previous results that specific PUFA profiles characterize the AT in lean or obese condition and correlates with the frequencies of total resident Treg and NKT-like cells and that ω3 and ω6 PUFA can influence the inflammatory status of AT (25, 26, 38), we then assessed whether these latter could influence the transcriptional program of visceral adipocytes, including the expression of genes deregulated in obesity. To this end, freshly isolated adipocytes from a subset of individuals within the lean and obese subject categories, were incubated with the anti-inflammatory ω3 PUFA DHA or pro-inflammatory ω6 PUFA AA, and the expression of a panel of genes previously selected for RNASeq validation was assessed 18 h later by real-time qPCR. As shown in Table 2, adipocytes from lean and obese individuals showed a different responsiveness to DHA and AA exposure. In particular, adipocytes from lean subjects exhibited a greater overall response to DHA, with most of the genes being up-regulated, compared to those from Ob subjects, where the number of DHA regulated genes was smaller and showed a general down-regulation. Conversely, a small number of genes were up-regulated in lean subjects after AA exposure, while obese individuals showed a greater response in terms of number of genes modulated and extent of induction. In particular, DHA up-regulated FADS1 and FADS2 in the lean subject category whose expression was found to decrease in Ob with respect to lean subjects (Table 2 and Figure 5). Likewise, DHA acted on genes like BMPER3, NFATC4 and ADAM15, belonging to categories deregulated under obesity adding further evidence for a potential role of this PUFA in attenuating obesity-related AT inflammation. Furthermore, the expression of CD36, ADIPOR2, ACACA and DDR1 was up-regulated by DHA in lean subjects. ACACA and DDR1, genes involved in the biosynthesis of FA and among other functions in the modulation of insulin signaling, showed an opposite modulation in Ob vs. Nw (down- and up-modulated, respectively). Conversely, AA modulated a different set of genes involved in ECM remodeling (SPP1, ECM1, LTBP2, TNC) in obese subjects, with the exception of CAST, that was up-regulated in both lean and obese subjects. Genes involved in AT inflammation (HP, IL6ST) and the proto-oncogene FYN were also up-modulated by AA in obese subjects.

**Table 2:** PUFA modulation of selected adipocyte genes in lean and obese subjects.

**Subjects group**	**FA treatment**					
		**DHA**			**Ob vs. Nw**	
	**Gene**	**FC**	***p*-value**	**Gene**	**FC**	***p*-value**
Normal weight	ACACA	4.06	0.002	ACACA	2.31	0.010
	PIK3CD	2.84	0.018	PIK3CD	0.64	ns
	ADIPOR2	2.41	0.017	ADIPOR2	1.32	ns
	FADS1	2.30	0.032	FADS1	0.45	0.000
	LPIN2	1.83	0.002	LPIN2	1.35	ns
	FADS2	1.80	0.000	FADS2	0.52	0.001
	CD36	1.77	0.017	CD36	2.55	0.050
	DDR1	1.56	0.017	DDR1	1.59	ns
	COL4A2	1.54	0.001	COL4A2	2.58	0.003
	AKT2	1.52	0.028	AKT2	1.50	0.001
	SMAD2	1.39	0.001	SMAD2	1.41	ns
	ANGPT1	0.50	0.029	ANGPT1	4.55	0.006
Obese	CD9	1.63	0.050	CD9	14.09	ns
	NFATC4	0.78	0.000	NFATC4	1.01	ns
	ACACA	0.69	0.050	ACACA	2.31	0.010
	DDR1	0.55	0.007	DDR1	1.59	ns
	ADAM15	0.26	0.039	ADAM15	6.58	ns
	BMP3	0.25	0.007	BMP3	1.87	ns
		**AA**			**Ob vs. Nw**	
	**Gene**	**FC**	***p*****-value**	**Gene**	**FC**	***p*****-value**
Normal weight	CAST	2.01	0.002	CAST	1.23	ns
	LTBP2	1.74	0.034	LTBP2	1.10	ns
	TNFSF10	0.40	0.009	TNFSF10	1.55	ns
Obese	TNC	7.58	0.028	TNC	0.65	ns
	CCND1	5.35	0.000	CCND1	1.85	ns
	SPP1	4.23	0.008	SPP1	14.63	ns
	HP	3.20	0.018	HP	1.02	ns
	IL6ST	2.65	0.050	IL6ST	1.11	ns
	FYN	2.13	0.039	FYN	1.87	ns
	ECM1	1.80	0.013	ECM1	0.83	ns
	CAST	1.67	0.007	CAST	1.23	ns

*In the left columns, results summary from the real-time qPCR analysis of lean and obese subjects after DHA and AA exposure (n = 3 subjects/category). In the right columns, results from the comparison between non treated adipocytes from obese and lean subjects are reported. ns, not significant*.

## Discussion

Obesity and CRC, whose prevalence is in constant increase, have become a very concerning public health issue. These multifactorial disorders/diseases are strongly interconnected and their relationship is also reinforced by the importance of nutrition especially in this type of cancer (39, 40). Despite the strong association of CRC with lifestyle factors and the identification of some biomarkers of CRC risk, the mechanisms underlying the higher susceptibility to cancer development and the poorer cancer prognosis documented for obese individuals are still a matter of debate. The correlation between obesity and CRC is strongly supported by epidemiological evidence, which clearly indicates that the risk of developing CRC is enhanced by obesity, leading to a worse prognosis after diagnosis and to increased mortality (2, 41, 42). Adipose tissue-associated inflammation, is nowadays recognized as an important determinant of the risk of developing obesity-related CRC. Specifically, VAT has an important role in the establishment of obesity-associated cancer due to its privileged localization to portal circulation and its capacity to secrete key bioactive substances (43). Although a direct relationship between obesity, AT dysfunction and CRC has been hypothesized in recent years, no definitive conclusions have been reached regarding the factors and processes involved therein. We approached this issue by analyzing the whole transcriptome of human visceral adipocytes by RNASeq, a powerful tool for studying the mechanisms underlying complex diseases. Of note, only few studies have evaluated the transcriptomic changes occurring in human adipocytes purified from VAT underlying obesity, as well as the effects of obesity or cancer status on their transcriptional program. Obesity-associated alterations of adipocyte transcriptome have been mainly investigated in whole SAT to assess the effects of bariatric surgery or low-calorie diets on gene expression (14, 20–22). In this regard it is noteworthy that in obesity the visceral fat is known to become more pro-inflammatory and is more directly linked to the risk of developing CRC rather than SAT, thus highlighting the importance of studying this fat compartment (22). In this study, we aimed to shed light on biological processes that are dysregulated in obesity and might contribute to set the basis for a more tumor-prone microenvironment. To the best of our knowledge this is the first study that performed a comparative transcriptomic analysis on human visceral adipocytes to assess how obesity, alone or combined to CRC, affects their transcriptional program, and to assess the influence of BMI on the adipocyte transcriptional program in CRC. Although the average age of CRC patients is higher than that of non-CRC subjects, the strong modulation of gene expression observed suggests that age-related differences may have exerted only a limited influence on the results achieved. Furthermore, the differences found between lean and obese subjects, as well as between lean subjects affected by CRC and obese subjects affected by CRC, suggest that the described obesity effects are irrespective of age.

Transcriptomic analysis revealed marked differences in the transcriptional program of adipocytes in obesity, with a large number of genes down-regulated rather than up-regulated. In keeping with the observed obesity-associated metabolic alterations, the dysregulated genes belonged to pathways and biological processes related to FA, glucose and pyruvate metabolism. In this regard, Klimcakova and colleagues found that the progression from lean to the obese state and further progression to metabolic syndrome was associated with a coordinated repression of fat cell metabolism in both VAT and SAT (18). Furthermore, Franck and colleagues reported that genes involved in lipogenesis, protein synthesis and insulin resistance are of critical importance in adipocyte response to changes in caloric intake (44). In this study, we extend this knowledge by providing a model in which the transition from a lean to an obese condition parallels a transcriptional reprogramming of fat cells and AT remodeling, through the modulation of inflammation (Adipocytokine signaling pathway, Platelet degranulation) and lipid metabolism (Pyruvate and glucose metabolism, PPAR and AMPK signaling pathway). Moreover, our results point to Pyruvate metabolism as central process in disease-induced adipocyte dysfunction, being shared between obesity and cancer-affected groups.

We also report that BMI has an impact on the transcriptional program of adipocytes under CRC condition as different pathways and biological processes are differently enriched in lean and obese subjects affected by CRC. Although obesity and CRC appear to affect different biological processes in visceral adipocytes when analyzed independently, cancer-induced transcriptional program is deeply affected by obesity, with adipocytes from Ob individuals exhibiting a more complex response to the tumor, in term of number of genes and processes found to be deregulated. Our results suggest the involvement of lipid metabolism, glucagon and insulin signaling pathways in obesity-driven alterations of adipocyte functions related to carcinogenesis. However, it remains to be determined whether these pathways may set the basis for the negative influence of obesity in disease progression, prognosis or response to therapy.

Obesity-associated AT modifications often resemble those observed within the tumor microenvironment (e.g., inflammation, leukocyte infiltration, hypoxia, angiogenesis). In obesity AT contributes to tumor initiation and progression by functioning as an endocrine organ, through the release of soluble mediators of cancer development like adipokines, pro-inflammatory cytokines, growth and proangiogenic factors, and ECM constituents, or metabolites acting as an energy reservoir for cancer cells (e.g., free FA, lactate), collectively providing a link between obesity and cancer (45). Furthermore, the ubiquitous distribution of AT in the body determines that many solid tumors grow in proximity or in close contact with adipocytes. Thus, cancer cells own the potential to reprogram adipocytes to support tumor growth (46).

RNASeq represents a valuable tool to reduce the complexity of and reveal biological processes/pathways in genome-wide expression studies and to narrow down the panel of genes to be further analyzed. However, the technical/statistical features of RNASeq output may lead to bias in the results when compared to other methods. In this study, we used RNASeq to define the general landscape of processes occurring in the different categories of subjects and refined the results by real-time qPCR. In consideration of the higher sensitivity of the latter, as well as of its capacity to quantify gene expression without discriminating among transcript variants with respect to RNASeq, we based our data interpretation on the results achieved by real-time qPCR analysis. In this context it is of interest that among genes deregulated under CRC conditions, the majority are involved in cell adhesion/migration processes and ECM remodeling, such as collagens (e.g., COL4A2, COL6A3), or genes encoding for structural and non-structural matricellular proteins (e.g., ECM2, SPARC and DDR1). In addition, the “*Extracellular matrix”* category resulted to be enriched when the genes shared between Ob and ObCRC as well as NwCRC and ObCRC subjects were considered (Figure 4). Of note, adipocyte function and survival is tightly regulated by both the molecular composition and mechanical properties of the ECM (47). Reduced tissue oxygenation induces changes in the transcriptional program of adipocytes leading to excessive deposition of ECM components, triggering adipocyte necrosis, attracting pro-inflammatory immune cells (19) and ultimately leading to fibrosis development (48). Indeed, AT of obese subjects exhibits a higher degree of fibrosis than lean subjects (49) and hypoxia-induced fibrosis is associated with the onset of metabolic perturbations and non-resolved inflammation in adipocytes (50, 51). Furthermore, it has been suggested that ECM remodeling occurring in obesity has important metabolic consequences, damaging impact on glucose homoeostasis and may contribute to insulin resistance (52, 53). In addition, interaction of adipose matrix molecules with their cognate receptors by a diverse array of AT cells further contributes to metabolic deteriorations in obesity (54). In particular, collagen components processed within the AT to produce pro-angiogenic factors, together with alterations of TGFβ signaling further favor AT vascularization, and inflammation (43, 55). In this regard, angiogenesis represents a key event in carcinogenesis and, in the transition from lean to obese state, is critical to promote AT accumulation (56). Accordingly, we found that genes related to angiogenesis (e.g., ANGPT1, ANXA2), as well as to TGFβ signaling (e.g., SMAD4, BMPER, AKT2) are modulated in obesity condition. Hence, obesity-induced AT ECM changes may provide a more favorable environment to developing tumors, thus providing a link between excessive adiposity and cancer.

Growing evidence highlights an association between metabolic factors and increased risk of colorectal carcinogenesis with the local metabolic alterations in AT in individuals with obesity resulting in multiple systemic metabolic alterations, such as insulin resistance, hyper-glycaemia, dyslipidemia and chronic inflammation (57). Accordingly, we found that genes belonging to pyruvate and FA metabolism, are dysregulated in both obesity and CRC. The observed alteration of genes encoding for key enzymes in these processes, such as PCK1 and PCK2, ALDH3A2, ALDH2, ALDH7A1, LDHB, LDHD, FADS1, FADS2, FADS5 and FASN may reprogram glucose metabolism and lipogenesis leading to AT dysmetabolism, and have profound pathophysiological effects to critically shape the tumor microenvironment. Finally, it is of interest that the majority of the genes shared by obesity and CRC are organized into small networks of functional interactions or enrich categories of biological processes that are related to metabolic processes, fibrosis and inflammation.

Although we did not observe any dysregulation of NLRP3 inflammasome-related genes previously described in SAT adipocytes or whole SAT (16, 17), inflammation-related genes were also found dysregulated in VAT adipocytes from obese and CRC subjects. It is also of interest that processes linked to platelet degranulation/activation are significantly modulated in all pathological conditions. In this regard, besides contributing to thrombosis, platelets have emerged as potent influencers of cancer-associated inflammation as upon activation, they release a plethora of factors that modulate the tumor microenvironment and can promote tumor progression, ECM remodeling and angiogenesis (58). Modulation of other inflammation-related pathways, such as the NFAT signaling cascade, known to be involved in inflammasome activation and in the regulation of adipokine transcription in AT of animal models (59, 60), and the CAPN10/CAST system, known to influence multiple functions including apoptosis, fibrosis and inflammation (61), and the FYN gene, shown to be an important regulator of inflammatory signaling in AT (62), was also observed in obesity and obesity-associated CRC conditions.

Based on our previous studies showing that PUFA endowed with pro- or anti-inflammatory properties modulates the adipocyte inflammatory status (26, 38), in this study we further investigated the effects of AA and DHA on adipocyte transcriptional program. The results achieved indicate that DHA and AA induce different transcriptional changes in adipocytes from lean vs. obese subjects. While DHA has a more pronounced capacity to up-regulate genes in lean adipocytes, the opposite is observed in obese subjects highlighting the BMI-dependent differential response of adipocytes to food components. Moreover, our results provide further evidence for a potential role of DHA in attenuating obesity-related AT inflammation since this PUFA exhibit the capacity to counteract the detrimental effects of obesity on some genes mainly related to metabolism and ECM remodeling. Of note, these genes were previously found to be modulated by weight loss achieved both by dietary or bariatric surgery interventions (20–22, 44, 63). In summary, our data provide further support for the central role of AT functional alterations in linking obesity to cancer. Obesity-associated transcriptional changes of visceral adipocytes may contribute to tumor promoting processes including inflammation, lipid and glucose metabolism, fibrosis, cell-cell communication, release of circulating factors to signal to other tissue and coordinate energy metabolism. Although computational analysis allows to identify candidate genes relevant to many biological process, their description is correlative and no causality can be directly established. Indeed, the relationship between obesity and CRC is complex and the comprehension of the underlying mechanisms requires further experimental and functional studies. Nonetheless, components of the interrelated processes and pathways unraveled by our study represent promising mechanism-based targets for breaking the links between obesity and its metabolic dysregulation, and cancer. In this scenario, the regulatory action of dietary PUFA on AT genomics adds further evidence for a role of diet in the modulation of AT inflammation, metabolism and ECM remodeling and for its application in cancer chemoprevention.

## Ethics Statement

Investigation has been conducted in accordance with the ethical standards and with the Declaration of Helsinki, and according to national and international guidelines. It has been approved by the authors' institutional Ethics Committee. All enrolled subjects were provided with complete information about the study and signed an informed consent.

## Author Contributions

AB, RV, BS, and MD prepared samples for RNASeq and performed real-time qPCR for gene validation. EC, PM, and CR performed RNASeq data analyses. AB and AM performed pathway analyses and real-time. AB, AM, EC, PM, and CR provided computational and statistical support throughout the study. MDC, LC, and SG provided substantial contributions to the conception of the work as well as interpretation of data and manuscript writing.

### Conflict of Interest Statement

The authors declare that the research was conducted in the absence of any commercial or financial relationships that could be construed as a potential conflict of interest.

## Abbreviations

AT: adipose tissue
AA: arachidonic acid
BMI: body mass index
CRC: colorectal cancer
DET: differentially expressed transcripts
DHA: docosahexaenoic acid
ECM: extracellular matrix
FA: fatty acids
Nw: normal weight
NwCRC: normal weight affected by CRC
Ob: obese
ObCRC: obese affected by CRC
PUFA: polyunsaturated fatty acids
SAT: subcutaneous adipose tissue
VAT: visceral adipose tissue.

## References

[1] WHO Obesity and Overweight. Fact sheet (2015). Available online at: http://www.who.int/mediacentre/factsheets/fs311/en/

[2] Bardou M Barkun AN Martel M. Obesity and colorectal cancer. Gut (2013) 62:933–47. 10.1136/gutjnl-2013-304701 23481261

[3] Park J Morley TS Kim M Clegg DJ Scherer PE. Obesity and cancer–mechanisms underlying tumour progression and recurrence. Nat Rev Endocrinol. (2014) 10:455–65. 10.1038/nrendo.2014.94 24935119 PMC4374431

[4] Unamuno X Gomez-Ambrosi J Rodriguez A Becerril S Fruhbeck G Catalan V. Adipokine dysregulation and adipose tissue inflammation in human obesity. Eur J Clin Invest. (2018) 48:e12997. 10.1111/eci.12997 29995306

[5] O'Sullivan J Lysaght J Donohoe CL Reynolds JV. Obesity and gastrointestinal cancer: the interrelationship of adipose and tumour microenvironments. Nat Rev Gastroenterol Hepatol. (2018) 15:699–714. 10.1038/s41575-018-0069-7 30323319

[6] Martinez-Gonzalez MA Zazpe I Razquin C Sanchez-Tainta A Corella D Salas-Salvado J. Empirically-derived food patterns and the risk of total mortality and cardiovascular events in the PREDIMED study. Clin Nutr. (2015) 34:859–67. 10.1016/j.clnu.2014.09.006 25304294

[7] Turati F Edefonti V Bravi F Ferraroni M Talamini R Giacosa A. Adherence to the European food safety authority's dietary recommendations and colorectal cancer risk. Eur J Clin Nutr. (2012) 66:517–22. 10.1038/ejcn.2011.217 22234042

[8] Campbell KL Landells CE Fan J Brenner DR. A systematic review of the effect of lifestyle interventions on adipose tissue gene expression: implications for carcinogenesis. Obesity (2017) 25 (Suppl. 2):S40–51. 10.1002/oby.22010 29086521

[9] Garaulet M Hernandez-Morante JJ Tebar FJ Zamora S. Relation between degree of obesity and site-specific adipose tissue fatty acid composition in a Mediterranean population. Nutrition (2011) 27:170–6. 10.1016/j.nut.2010.01.004 20541362

[10] Rogero MM Calder PC. Obesity, inflammation, toll-like receptor 4 and fatty acids. Nutrients (2018) 10:E432. 10.3390/nu10040432 29601492 PMC5946217

[11] Masoodi M Kuda O Rossmeisl M Flachs P Kopecky J. Lipid signaling in adipose tissue: connecting inflammation & metabolism. Biochim Biophys Acta (2015) 1851:503–18. 10.1016/j.bbalip.2014.09.023 25311170

[12] Khadge S Sharp JG McGuire TR Thiele GM Black P DiRusso C. Immune regulation and anti-cancer activity by lipid inflammatory mediators. Int Immunopharmacol. (2018) 65:580–92. 10.1016/j.intimp.2018.10.026 30447537 PMC6322683

[13] Martinez-Useros J Garcia-Foncillas J. Obesity and colorectal cancer: molecular features of adipose tissue. J Transl Med. (2016) 14:21. 10.1186/s12967-016-0772-5 26801617 PMC4722674

[14] Huerta AE Prieto-Hontoria PL Fernandez-Galilea M Escote X Martinez JA Moreno-Aliaga MJ. Effects of dietary supplementation with EPA and/or alpha-lipoic acid on adipose tissue transcriptomic profile of healthy overweight/obese women following a hypocaloric diet. Biofactors (2017) 43:117–31. 10.1002/biof.1317 27507611

[15] Li J Zhou C Li J Su Z Sang H Jia E. Global correlation analysis for microRNA and gene expression profiles in human obesity. Pathol Res Pract. (2015) 211:361–8. 10.1016/j.prp.2014.11.014 25701361

[16] Yin Z Deng T Peterson LE Yu R Lin J Hamilton DJ. Transcriptome analysis of human adipocytes implicates the NOD-like receptor pathway in obesity-induced adipose inflammation. Mol Cell Endocrinol. (2014) 394:80–7. 10.1016/j.mce.2014.06.018 25011057 PMC4219530

[17] Goossens GH Blaak EE Theunissen R Duijvestijn AM Clement K Tervaert JW. Expression of NLRP3 inflammasome and T cell population markers in adipose tissue are associated with insulin resistance and impaired glucose metabolism in humans. Mol Immunol. (2012) 50:142–9. 10.1016/j.molimm.2012.01.005 22325453

[18] Klimcakova E Roussel B Marquez-Quinones A Kovacova Z Kovacikova M Combes M. Worsening of obesity and metabolic status yields similar molecular adaptations in human subcutaneous and visceral adipose tissue: decreased metabolism and increased immune response. J Clin Endocrinol Metab. (2011) 96:E73–82. 10.1210/jc.2010-1575 21047918

[19] Henegar C Tordjman J Achard V Lacasa D Cremer I Guerre-Millo M. Adipose tissue transcriptomic signature highlights the pathological relevance of extracellular matrix in human obesity. Genome Biol. (2008) 9:R14. 10.1186/gb-2008-9-1-r14 18208606 PMC2395253

[20] Armenise C Lefebvre G Carayol J Bonnel S Bolton J Di Cara A. Transcriptome profiling from adipose tissue during a low-calorie diet reveals predictors of weight and glycemic outcomes in obese, nondiabetic subjects. Am J Clin Nutr. (2017) 106:736–46. 10.3945/ajcn.117.156216 28793995

[21] Ferguson JF Xue C Hu Y Li M Reilly MP. Adipose tissue RNASeq reveals novel gene-nutrient interactions following n-3 PUFA supplementation and evoked inflammation in humans. J Nutr Biochem. (2016) 30:126–32. 10.1016/j.jnutbio.2015.12.010 27012629 PMC4808243

[22] Poitou C Perret C Mathieu F Truong V Blum Y Durand H. Bariatric surgery induces disruption in inflammatory signaling pathways mediated by immune cells in adipose tissue: a RNA-Seq study. PLoS ONE (2015) 10:e0125718. 10.1371/journal.pone.0125718 25938420 PMC4418598

[23] Despres JP Lemieux I. Abdominal obesity and metabolic syndrome. Nature (2006) 444:881–7. 10.1038/nature05488 17167477

[24] Oh TH Byeon JS Myung SJ Yang SK Choi KS Chung JW. Visceral obesity as a risk factor for colorectal neoplasm. J Gastroenterol Hepatol. (2008) 23:411–7. 10.1111/j.1440-1746.2007.05125.x 17725596

[25] Donninelli G Del Corno M Pierdominici M Scazzocchio B Vari R Varano B. Distinct blood and visceral adipose tissue regulatory T cell and innate lymphocyte profiles characterize obesity and colorectal cancer. Front Immunol. (2017) 8:643. 10.3389/fimmu.2017.00643 28649243 PMC5465245

[26] Del Corno M D'Archivio M Conti L Scazzocchio B Vari R Donninelli G. Visceral fat adipocytes from obese and colorectal cancer subjects exhibit distinct secretory and omega6 polyunsaturated fatty acid profiles and deliver immunosuppressive signals to innate immunity cells. Oncotarget (2016) 7:63093–105. 10.18632/oncotarget.10998 27494857 PMC5325349

[27] Kim D Langmead B Salzberg SL. HISAT: a fast spliced aligner with low memory requirements. Nat Methods (2015) 12:357–60. 10.1038/nmeth.3317 25751142 PMC4655817

[28] Pertea M Pertea GM Antonescu CM Chang TC Mendell JT Salzberg SL. StringTie enables improved reconstruction of a transcriptome from RNA-seq reads. Nat Biotechnol. (2015) 33:290–5. 10.1038/nbt.3122 25690850 PMC4643835

[29] Robinson MD McCarthy DJ Smyth GK. edgeR: a bioconductor package for differential expression analysis of digital gene expression data. Bioinformatics (2010) 26:139–40. 10.1093/bioinformatics/btp616 19910308 PMC2796818

[30] Huang DW Sherman BT Tan Q Kir J Liu D Bryant D. DAVID bioinformatics resources: expanded annotation database and novel algorithms to better extract biology from large gene lists. Nucleic Acids Res. (2007) 35:W169–75. 10.1093/nar/gkm415 17576678 PMC1933169

[31] Warde-Farley D Donaldson SL Comes O Zuberi K Badrawi R Chao P. The GeneMANIA prediction server: biological network integration for gene prioritization and predicting gene function. Nucleic Acids Res. (2010) 38:W214–20. 10.1093/nar/gkq537 20576703 PMC2896186

[32] Tarasiuk A Mosinska P Fichna J. The mechanisms linking obesity to colon cancer: an overview. Obes Res Clin Pract. (2018) 12:251–9. 10.1016/j.orcp.2018.01.005 29428365

[33] Hanahan D Weinberg RA. Hallmarks of cancer: the next generation. Cell (2011) 144:646–74. 10.1016/j.cell.2011.02.013 21376230

[34] Menendez JA Lupu R. Fatty acid synthase and the lipogenic phenotype in cancer pathogenesis. Nat Rev Cancer (2007) 7:763–77. 10.1038/nrc2222 17882277

[35] Huang CW Chien YS Chen YJ Ajuwon KM Mersmann HM Ding ST. Role of n-3 polyunsaturated fatty acids in ameliorating the obesity-induced metabolic syndrome in animal models and humans. Int J Mol Sci. (2016) 17:E1689. 10.3390/ijms17101689 27735847 PMC5085721

[36] Pan P Yu J Wang LS. Colon cancer: what we eat. Surg Oncol Clin N Am. (2018) 27:243–67. 10.1016/j.soc.2017.11.002 29496088 PMC5836483

[37] Miccadei S Masella R Mileo AM Gessani S. omega3 polyunsaturated fatty acids as immunomodulators in colorectal cancer: new potential role in adjuvant therapies. Front Immunol. (2016) 7:486. 10.3389/fimmu.2016.00486 27895640 PMC5108786

[38] D'Archivio M Scazzocchio B Giammarioli S Fiani ML Vari R Santangelo C. omega3-PUFAs exert anti-inflammatory activity in visceral adipocytes from colorectal cancer patients. PLoS ONE (2013) 8:e77432. 10.1371/journal.pone.0077432 24116229 PMC3792028

[39] Mathers JC. Obesity and bowel cancer: from molecular mechanisms to interventions. Nutr Res. (2018). [Epub ahead of print]. 10.1016/j.nutres.2018.08.004 30274687

[40] Huxley RR Ansary-Moghaddam A Clifton P Czernichow S Parr CL Woodward M. The impact of dietary and lifestyle risk factors on risk of colorectal cancer: a quantitative overview of the epidemiological evidence. Int J Cancer (2009) 125:171–80. 10.1002/ijc.24343 19350627

[41] Comstock SS Hortos K Kovan B McCaskey S Pathak DR Fenton JI. Adipokines and obesity are associated with colorectal polyps in adult males: a cross-sectional study. PLoS ONE (2014) 9:e85939. 10.1371/journal.pone.0085939 24465801 PMC3895019

[42] Park H Kim M Kwon GT Lim DY Yu R Sung MK. A high-fat diet increases angiogenesis, solid tumor growth, and lung metastasis of CT26 colon cancer cells in obesity-resistant BALB/c mice. Mol Carcinog. (2012) 51:869–80. 10.1002/mc.20856 21919080

[43] Alves MG Moreira A Guimaraes M Nora M Sousa M Oliveira PF. Body mass index is associated with region-dependent metabolic reprogramming of adipose tissue. BBA Clin. (2017) 8:1–6. 10.1016/j.bbacli.2017.05.001 28567337 PMC5440253

[44] Franck N Gummesson A Jernas M Glad C Svensson PA Guillot G. Identification of adipocyte genes regulated by caloric intake. J Clin Endocrinol Metab. (2011) 96:E413–8. 10.1210/jc.2009-2534 21047925

[45] Himbert C Delphan M Scherer D Bowers LW Hursting S Ulrich CM. Signals from the adipose microenvironment and the obesity-cancer link-a systematic review. Cancer Prev Res. (2017) 10:494–506. 10.1158/1940-6207.CAPR-16-0322 28864539 PMC5898450

[46] Zoico E Rizzatti V Darra E Budui SL Franceschetti G Vinante F. Morphological and functional changes in the peritumoral adipose tissue of colorectal cancer patients. Obesity (2017) 25 (Suppl. 2):S87–94. 10.1002/oby.22008 29086519

[47] Mariman EC Wang P. Adipocyte extracellular matrix composition, dynamics and role in obesity. Cell Mol Life Sci. (2010) 67:1277–92. 10.1007/s00018-010-0263-4 20107860 PMC2839497

[48] Sun K Tordjman J Clement K Scherer PE. Fibrosis and adipose tissue dysfunction. Cell Metab. (2013) 18:470–7. 10.1016/j.cmet.2013.06.016 23954640 PMC3795900

[49] Divoux A Tordjman J Lacasa D Veyrie N Hugol D Aissat A. Fibrosis in human adipose tissue: composition, distribution, and link with lipid metabolism and fat mass loss. Diabetes (2010) 59:2817–25. 10.2337/db10-0585 20713683 PMC2963540

[50] Datta R Podolsky MJ Atabai K Fat fibrosis: friend or foe? JCI Insight (2018) 3:122289 10.1172/jci.insight.122289 30282827 PMC6237440

[51] Khan T Muise ES Iyengar P Wang ZV Chandalia M Abate N. Metabolic dysregulation and adipose tissue fibrosis: role of collagen VI. Mol Cell Biol. (2009) 29:1575–91. 10.1128/MCB.01300-08 19114551 PMC2648231

[52] Williams AS Trefts E Lantier L Grueter CA Bracy DP James FD. Integrin-linked kinase is necessary for the development of diet-induced hepatic insulin resistance. Diabetes (2017) 66:325–34. 10.2337/db16-0484 27899483 PMC5248997

[53] Barreto SC Hopkins CA Bhowmick M Ray A. Extracellular matrix in obesity - cancer interactions. Horm Mol Biol Clin Investig. (2015) 22:63–77. 10.1515/hmbci-2015-0001 25870970

[54] Lin Chun TH Kang L. Adipose extracellular matrix remodelling in obesity and insulin resistance. Biochem Pharmacol. (2016) 119:8–16. 10.1016/j.bcp.2016.05.005 27179976 PMC5061598

[55] Karsdal MA Nielsen SH Leeming DJ Langholm LL Nielsen MJ Manon-Jensen T. The good and the bad collagens of fibrosis-Their role in signaling and organ function. Adv Drug Deliv Rev. (2017) 121:43–56. 10.1016/j.addr.2017.07.014 28736303

[56] Crewe C An YA Scherer PE. The ominous triad of adipose tissue dysfunction: inflammation, fibrosis, and impaired angiogenesis. J Clin Invest. (2017) 127:74–82. 10.1172/JCI88883 28045400 PMC5199684

[57] Yehuda-Shnaidman E Schwartz B. Mechanisms linking obesity, inflammation and altered metabolism to colon carcinogenesis. Obes Rev. (2012) 13:1083–95. 10.1111/j.1467-789X.2012.01024.x 22937964

[58] Olsson AK Cedervall J. The pro-inflammatory role of platelets in cancer. Platelets (2018) 29:569–73. 10.1080/09537104.2018.1453059 29584534

[59] Ahmad F Chung YW Tang Y Hockman SC Liu S Khan Y. Phosphodiesterase 3B (PDE3B) regulates NLRP3 inflammasome in adipose tissue. Sci Rep. (2016) 6:28056. 10.1038/srep28056 27321128 PMC4913246

[60] Omar B Banke E Guirguis E Akesson L Manganiello V Lyssenko V Regulation of the pro-inflammatory cytokine osteopontin by GIP in adipocytes–a role for the transcription factor NFAT and phosphodiesterase 3B. Biochem Biophys Res Commun. (2012) 425:812–7. 10.1016/j.bbrc.2012.07.157 22892131 PMC3759516

[61] Muniappan L Javidan A Jiang W Mohammadmoradi S Moorleghen JJ Katz WS. Calpain inhibition attenuates adipose tissue inflammation and fibrosis in diet-induced obese mice. Sci Rep. (2017) 7:14398. 10.1038/s41598-017-14719-9 29089532 PMC5663911

[62] Lee TW Kwon H Zong H Yamada E Vatish M Pessin JE. Fyn deficiency promotes a preferential increase in subcutaneous adipose tissue mass and decreased visceral adipose tissue inflammation. Diabetes (2013) 62:1537–46. 10.2337/db12-0920 23321073 PMC3636609

[63] Dahlman I Linder K Arvidsson Nordstrom E Andersson I Liden J Verdich C. Changes in adipose tissue gene expression with energy-restricted diets in obese women. Am J Clin Nutr. (2005) 81:1275–85. 10.1093/ajcn/81.6.1275 15941876

