# Supplementary material for: Transcriptome Profiles of Human Visceral Adipocytes in Obesity and Colorectal Cancer Unravel the Effects of Body Mass Index and Polyunsaturated Fatty Acids on Genes and Biological Processes Related to Tumorigenesis
Source: Front Immunol. 2019 Feb 19;10:265. doi: 10.3389/fimmu.2019.00265 (PMC6389660; doi:10.3389/fimmu.2019.00265)
Supplement: Supplementary file 3 [file Data_Sheet_3.pdf]

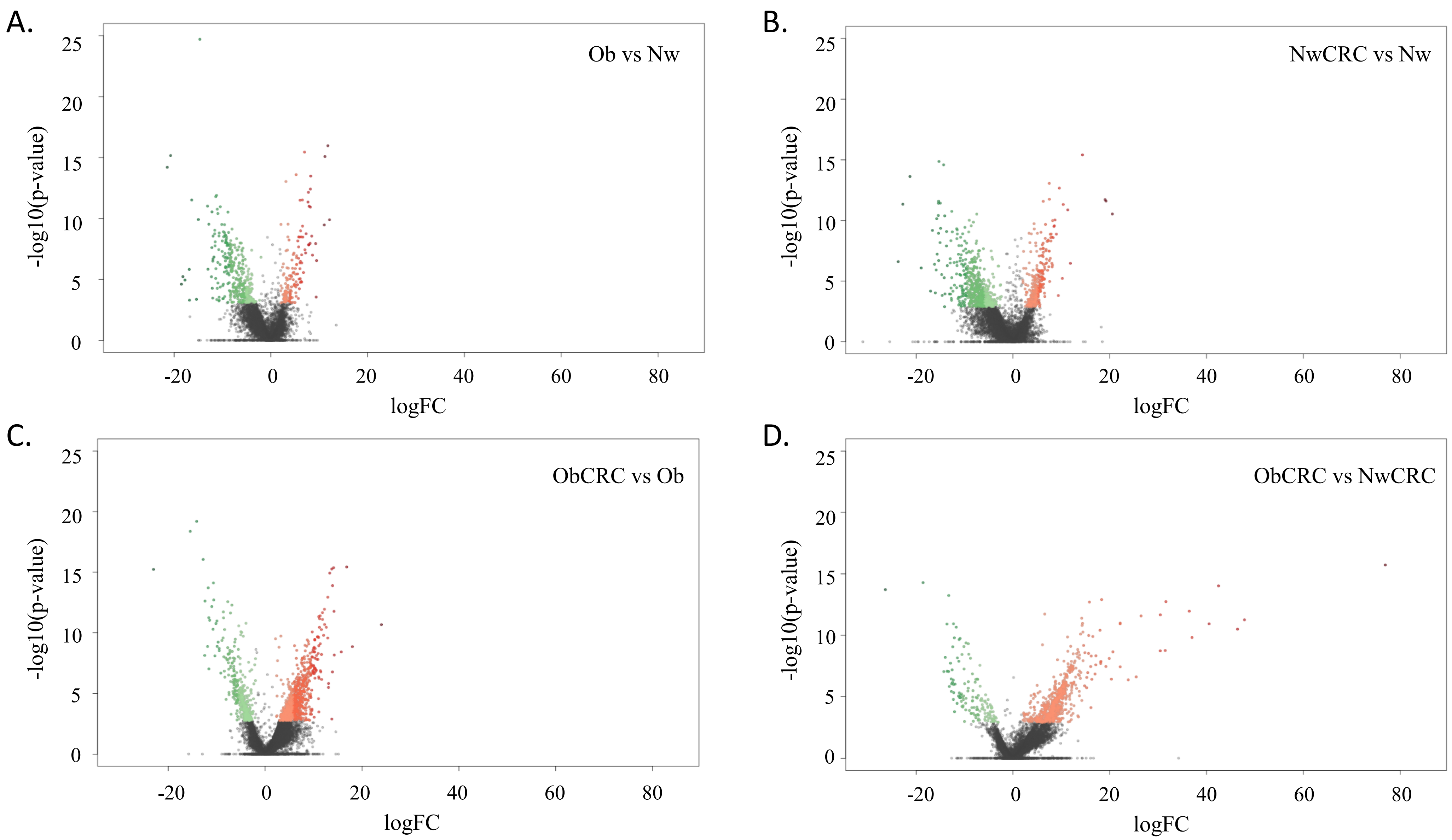

Supplemental Data 3: Volcano plots of differentially expressed transcripts. Scatter-plots of significance [-log10(p-value)] versus fold-change [log2(FC)], also called Volcano plots, of the four differentially expressed comparisons: (A) obese (Ob) vs normal weight (Nw); (B) normal weight affected by CRC (NwCRC) vs normal weight (Nw); (C) obese affected by CRC (ObCRC) vs obese (Ob); (D) obese affected by CRC (ObCRC) vs normal weight affected by CRC (NwCRC). Colored points represent differentially expressed transcripts (FDR lower than 0,05 and logFCgreater than |2|). Red and green shades (up- and down-modulated transcripts) are proportional to the log(FC) absolute values.
