# Supplementary material for: Transcriptome Profiles of Human Visceral Adipocytes in Obesity and Colorectal Cancer Unravel the Effects of Body Mass Index and Polyunsaturated Fatty Acids on Genes and Biological Processes Related to Tumorigenesis
Source: Front Immunol. 2019 Feb 19;10:265. doi: 10.3389/fimmu.2019.00265 (PMC6389660; doi:10.3389/fimmu.2019.00265)
Supplement: Supplementary file 6 [file Data_Sheet_6.docx]

**Supplemental Data 6:** Selected categories and associated genes shared between conditions or “unique” for each group resulting from KEGG pathway and GO enrichment analysis. Normal weight (Nw), obese (Ob), normal weight affected by CRC (NwCRC) and obese affected by CRC (ObCRC) groups.

| **Condition** | **Categories** | **DET** | **FDR** |
| --- | --- | --- | --- |
| **SHARED** |  |  |  |
| Ob and NwCRC groups | Pyruvate metabolism | ALDH3A2, ALDH7A1, LDHD | 0,0090 |
|  | Cellular aldehyde metabolic process | ALDH3A2, ALDH7A1, LDHD | 0,0478 |
|  | Extracellular exosome | BMP3, TM7SF3, PEPD, LTBP2, STK25, TBC1D10A, ACTN1, RFNG, ECM1, ALDH3A2, CD9, PSMA1, ALDH7A1, LAT2, HPGD, AUP1, MEST | 0,0105 |
|  | Regulation of apoptotic process | BMP3, FYN, SRA1, ACTN1 | 0,0478 |
| Ob and ObCRC groups | Pyruvate metabolism | LDHB, ALDH7A1, LDHD, ACACB | 0,0020 |
|  | Extracellular matrix | CRIP2, LMCD1, COL12A1, ECM1 | 0,0329 |
|  | Calcineurin-NFATsignaling cascade | NFATC2, NFATC1 LMCD1, IGF1 | 0,0477 |
| ObCRC and NwCRC groups | Fatty acid degradation | ACADVL, ALDH7A1, ADH1B | 0,0353 |
|  | Extracellular matrix | ACTG1, P4HB, RPS18, LTBP2, LMNA, MFGE8, ANXA2 | 0,0051 |
|  | Focal adhesion | ACTG1, CD9, P4HB, RPS18, GNB2, AIF1L, ACTN1 | 0,0135 |
|  | Cell-cell adhesion | CAST, TBC1D10A, NOP56, PPP1R13L, ANXA2 | 0,0303 |
|  | Extracellular exosome | NACA, TM7SF3, LTBP2, LGMN, CD53, ACTG1, CD9, N4BP2L2, AIF1L, TPT1, MEST, P4HB, PEPD, TBC1D10A, ARHGEF18, ACTN1, MFGE8, RFNG, ANXA2, APRT, MGAT1, TNFSF10, ALDH7A1, RPS18, GNB2, ADAM15 | 0,0008 |
|  | Apoptotic process | PDCL3, TNFSF10, KIF1B, TMEM214, SRA1, PPP1R13L, MRPS30, ADAM15 | 0,0191 |
| **“UNIQUE”** |  |  |  |
| NwCRC | Metabolic pathways | SAT1, PGS1, TUSC3, TALDO1, CYP2S1, ADH1B, CTPS2, OCRL, PFAS, AFMID, ALDH1A2, UPRT, LIAS, HSD17B4, TSTA3, PCYT2, GCNT1, PIGA, PLD3, POLR1D, PI4KA, AK2, AMPD2, POLR2J3, APRT, PCK1, MGAT1, MAN2A2, ACADVL, CTH, MPI, NNT, NDUFV1, POLD1, PGM1, ATP5A1, PHYKPL | 0,0412 |
|  | Transforming growth factor beta signaling pathway | ARHGEF18, SMAD6, CREB1, TGFBR2, TGFB3, ACVR1, CITED2, | 0,0277 |
|  | Extracellular/interstitial matrix | P4HB, ADAMTSL4, LMNA, TGFB3, MFGE8, DCN, DDX5, ANXA2, ACTG1, OGN, RPS18, LAMB2, RPS16, DSP, ATP5A1, TGFB1I1, TPSAB1, ECM2, SMOC2, | 0,0035 |
|  | Extracellular exosome | CHMP3, LGMN, IQGAP2, CD53, TTN, ACTG1, OGN, HSPH1, N4BP2L2, VPS13D, TMEM59, TPT1, AIF1L, SERPINA3, ANGPT1, CFI, FCGR3A, IAH1, PLD3, ARHGEF18, PI4KA, MFGE8, HLA-B, RFTN1, CTSV, TYK2, MGAT1, RPS18, CTH, IGSF8, RPS16, GNB2, VAMP8, PGM1, CYBRD1, DSP, CTSB, ADAM15, PAM, YWHAZ, TALDO1, NACA, NAPG, ABHD6, OCRL, ACP1, PFAS, ISLR, ANXA6, P3H1, LAMB2, NHLRC3, HSPA2, SH3GLB1, CNN2, HSPA4, C2, TSTA3, PHLDA3, ETFA, LY75, P4HB, RNASE1, PLA2G15, LRRN4, PSAP, ATRN, AK2, DDX5, LGALS9, APRT, PCK1, ANXA2, DUSP3, PSMC6, NRF1, TNFSF10, MPI, CPNE1, HGS, ATP5A1, MUC16 | 0,0077 |
|  | Cell-cell adhesion | CAST, YWHAZ, EPB41L1, SH3GLB1, PI4KA, KTN1, EXOC3, CNN2, NOP56, STAT1, ANXA2, GOLGA2 | 0,0412 |
| Ob | AMPK signaling pathway | LEP, CD36, STK11, IGF1, TBC1D1, RAB10, SREBF1, PFKFB3, HMGCR, PPP2R5D, PPP2R5C, ACACA, IGF1, STRADB, PCK2, ADIPOQ, LIPE, PIK3R1, AKT2 | 0,0482 |
|  | PPAR signaling | CD36, SLC27A6, SCP2, ACOX3 | 0,0482 |
|  | Adipose tissue development | LEP, PAXIP1, SPG20 | 0,0482 |
|  | Positive regulation of glucose import | CAPN10, IGF1, RHOQ | 0,0482 |
|  | Cellular response to insulin stimulus | CAPN10, SLC25A33, DENND4C, RHOQ, RAB10 | 0,0482 |
|  | Regulation of angiogenesis | LEP, ENPP2, RNH1 | 0,0482 |
|  | Adipocytokine signaling pathway | LEP, CD36, STK11, ADIPOR2 | 0,0482 |
| ObCRC | Regulation of lipolysis in adipocytes | ADCY3, GNAS, PNPLA2, PIK3R1, LIPE, AKT2 | 0,0485 |
|  | PI3K-Akt signaling pathway | CSF3, FGFR1, COL4A2, PDGFB, CREB1, PPP2R5D, PPP2R5C, PCK2, YWHAE, HSP90B1, GNB2, PRLR, ITGAV, ITGA8, MDM2, NOS3, MLST8, PIK3R1, AKT2 | 0,0496 |
|  | AMPK signaling pathway | SREBF1, PFKFB3, HMGCR, CREB1, PPP2R5D, PPP2R5C, ACACA, STRADB, PCK2, ADIPOQ, LIPE, PIK3R1, AKT2 | 0,0485 |
|  | Angiogenesis | COL18A1, FGFR1, COL4A2, ACVRL1, ACKR3, MFGE8, ANXA2, PDCL3, TYMP, HOXA3, ITGAV, NOS3, TNFSF12-TNFSF13, TMEM100, ADAM15 | 0,0485 |
|  | Negative regulation of apoptotic process | IER3, GCLC, IL6ST, UNG, NCKAP1L, ASNS, SOD2, MIF, IFIT3, SON, RPS6KA3, HSP90B1, BAG1, PRLR, PSEN2, TPT1, MDM2, NAIP, OGG1, PIK3R1, CYR61 | 0,0485 |
|  | Wnt signaling pathway | CSNK1D, PIAS4, CSNK2B, LEO1, CTNND1, RNF138, APCDD1, FBXW11, CCAR2, MARK1, BCL7B | 0,0485 |
|  | Cellular response to glucose starvation | ATG14, PIK3C3, ASNS, MYBBP1A | 0,0485 |
|  | Extracellular matrix | COL18A1, P4HB, COL4A2, PDGFB, LMNA, MFGE8, HNRNPU, ANXA2, ACTG1, HNRNPM, LGALS3BP, RPS18, HSP90B1, SERPINF1, RPL9, EIF4A1, RPS13, ATP5O, CYR61 COL18A1, DDR1, COL4A2, PDGFB, ITGAV, ITGA8, NF1, NID2, LOX, SPARC, CYR61 | 0,0485 |
|  | Collagen binding | DDR1, PDGFB, C1QTNF1, NID2, SPARC, PCOLCE2 | 0,0485 |
