# Supplementary material for: Transcriptome Profiles of Human Visceral Adipocytes in Obesity and Colorectal Cancer Unravel the Effects of Body Mass Index and Polyunsaturated Fatty Acids on Genes and Biological Processes Related to Tumorigenesis
Source: Front Immunol. 2019 Feb 19;10:265. doi: 10.3389/fimmu.2019.00265 (PMC6389660; doi:10.3389/fimmu.2019.00265)
Supplement: Supplementary file 7 [file Data_Sheet_7.docx]

**Supplemental Data 7:** List of genes and probes selected for real-time qPCR analysis.

| **Gene Symbol** | **Gene Name** | **Assay ID** |
| --- | --- | --- |
| ACACA | acetyl-CoA carboxylase alpha | Hs01046047_m1 |
| ACACB | acetyl-CoA carboxylase beta | Hs01565914_m1 |
| ACVRL1 | activin A receptor like type 1 | Hs00953798_m1 |
| ADAMTSL4 | ADAMTS like 4 | Hs00417524_m1 |
| ADIPOQ | adiponectin, C1Q and collagen domain containing | Hs00605917_m1 |
| ADIPOR2 | adiponectin receptor 2 | Hs00226105_m1 |
| AKT2 | AKT serine/threonine kinase 2 | Hs01086099_m1 |
| ALDH2 | aldehyde dehydrogenase 2 family (mitochondrial) | Hs01007998_m1 |
| ALDH3A2 | aldehyde dehydrogenase 3 family member A2 | Hs01116403_m1 |
| ALDH7A1 | aldehyde dehydrogenase 7 family member A1 | Hs00609622_m1 |
| ANGPT1 | angiopoietin 1 | Hs00919202_m1 |
| ANXA2 | annexin A2 | Hs01561520_m1 |
| ANXA6 | annexin A6 | Hs01049082_m1 |
| ANXA8L1;ANXA8 | annexin A8; annexin A8-like 1 | Hs00179940_m1 |
| ARNTL | aryl hydrocarbon receptor nuclear translocator like | Hs00154147_m1 |
| BMP3 | bone morphogenetic protein 3 | Hs00609638_m1 |
| BMPER | BMP binding endothelial regulator | Hs00403062_m1 |
| C1QTNF1 | C1q and tumor necrosis factor related protein 1 | Hs00945123_m1 |
| CALM3 | calmodulin 3 | Hs00270914_m1 |
| CAPN10 | calpain 10 | Hs01550167_m1 |
| CAST | calpastatin | Hs00156280_m1 |
| CCL20 | C-C motif chemokine ligand 20 | Hs00355476_m1 |
| CCND1 | cyclin D1 | Hs00765553_m1 |
| CD36 | CD36 molecule | Hs00354519_m1 |
| CD9 | CD9 molecule | Hs01124022_m1 |
| CITED2 | Cbp/p300 interacting transactivator with Glu/Asp rich carboxy-terminal domain 2 | Hs00366696_m1 |
| COL12A1 | collagen type XII alpha 1 chain | Hs00189184_m1 |
| COL18A1 | collagen type XVIII alpha 1 chain | Hs00181017_m1 |
| COL4A2 | collagen type IV alpha 2 chain | Hs00300500_m1 |
| COL6A3 | collagen type VI alpha 3 chain | Hs00915125_m1 |
| DDR1 | discoidin domain receptor tyrosine kinase 1 | Hs01058430_m1 |
| ECM1 | extracellular matrix protein 1 | Hs00189435_m1 |
| ECM2 | extracellular matrix protein 2 | Hs00154821_m1 |
| FADS1 | fatty acid desaturase 1 | Hs01096545_m1 |
| FADS2 | fatty acid desaturase 2 | Hs00927433_m1 |
| FADS5 | stearoyl-CoA desaturase | Hs01682761_m1 |
| FASN | fatty acid synthase | Hs01005622_m1 |
| FAT1 | FAT atypical cadherin 1 | Hs00170627_m1 |
| FGF1 | fibroblast growth factor 1 | Hs01092738_m1 |
| FOS | Fos proto-oncogene, AP-1 transcription factor subunit | Hs00170630_m1 |
| FYN | FYN proto-oncogene, Src family tyrosine kinase | Hs00176628_m1 |
| GNAS | GNAS complex locus | Hs00255603_m1 |
| HP | haptoglobin | Hs00978377_m1 |
| IGF1 | insulin like growth factor 1 | Hs01547656_m1 |
| IL32 | interleukin 32 | Hs00992441_m1 |
| IL6ST | interleukin 6 signal transducer | Hs00174360_m1 |
| ILF3 | interleukin enhancer binding factor 3 | Hs01128097_m1 |
| ITGAV | integrin subunit alpha V | Hs00233808_m1 |
| LDHB | lactate dehydrogenase B | Hs00929956_m1 |
| LEP | leptin | Hs00174877_m1 |
| LEPR;LEPROT | leptin receptor; leptin receptor overlapping transcript | Hs00213886_m1 |
| LIPE | lipase E, hormone sensitive type | Hs00943410_m1 |
| LITAF | lipopolysaccharide induced TNF factor | Hs01556090_m1 |
| LPIN2 | lipin 2 | Hs00206237_m1 |
| LTBP2 | latent transforming growth factor beta binding protein 2 | Hs00166367_m1 |
| MKL1 | megakaryoblastic leukemia (translocation) 1 | Hs00252979_m1 |
| MTHFR | methylenetetrahydrofolate reductase | Hs01114487_m1 |
| NFATC1 | nuclear factor of activated T-cells 1 | Hs00542675_m1 |
| NFATC2 | nuclear factor of activated T-cells 2 | Hs00905451_m1 |
| NFATC4 | nuclear factor of activated T-cells 4 | Hs00190037_m1 |
| NOTCH4 | notch 4 | Hs00965889_m1 |
| OPTN | optineurin | Hs00184221_m1 |
| PCK1 | phosphoenolpyruvate carboxykinase 1 | Hs00159918_m1 |
| PCK2 | phosphoenolpyruvate carboxykinase 2, mitochondrial | Hs00388934_m1 |
| PDGFB | platelet derived growth factor subunit B | Hs00966522_m1 |
| PDK2 | pyruvate dehydrogenase kinase 2 | Hs00176865_m1 |
| PDK4 | pyruvate dehydrogenase kinase 4 | Hs01037712_m1 |
| PDP1 | pyruvate dehyrogenase phosphatase catalytic subunit 1 | Hs00372607_m1 |
| PTEN | phosphatase and tensin homolog | Hs02621230_s1 |
| PIK3C3 | phosphatidylinositol 3-kinase catalytic subunit type 3 | Hs00176908_m1 |
| PIK3CD | phosphatidylinositol-4,5-bisphosphate 3-kinase catalytic subunit delta | Hs00192399_m1 |
| PIK3R1 | phosphoinositide-3-kinase regulatory subunit 1 | Hs00933163_m1 |
| PLIN1 | perilipin 1 | Hs00160173_m1 |
| RBP4 | retinol binding protein 4 | Hs00924047_m1 |
| SERPINB2 | serpin family B member 2 | Hs01010736_m1 |
| SERPINF1 | serpin family F member 1 | Hs01106937_m1 |
| SLC2A5 | solute carrier family 2 member 5 | Hs01086390_m1 |
| SMAD2 | SMAD family member 2 | Hs00998187_m1 |
| SMAD4 | SMAD family member 4 | Hs00929647_m1 |
| SMAD6 | SMAD family member 6 | Hs00178579_m1 |
| SOD2 | superoxide dismutase 2, mitochondrial | Hs00167309_m1 |
| SPARC | secreted protein acidic and cysteine rich | Hs00234160_m1 |
| SPP1 | secreted phosphoprotein 1 | Hs00959010_m1 |
| SREBF1 | sterol regulatory element binding transcription factor 1 | Hs01088691_m1 |
| TGFB1 | transforming growth factor beta 1 | Hs00998133_m1 |
| TGFBR2 | transforming growth factor beta receptor 2 | Hs00234253_m1 |
| TGFBR3 | transforming growth factor beta receptor 3 | Hs00234257_m1 |
| TNC | tenascin C | Hs01115664_m1 |
| TNFSF10 | tumor necrosis factor superfamily member 10 | Hs00921974_m1 |
| TTN | titin | Hs00399225_m1 |
| VASP | vasodilator-stimulated phosphoprotein | Hs01100128_m1 |
| VEGFA | vascular endothelial growth factor A | Hs00900055_m1 |
